# A Formalism for Evaluating Analytically the Cross-Correlation Structure of a Firing-Rate Network Model

**DOI:** 10.1186/s13408-015-0020-y

**Published:** 2015-03-15

**Authors:** Diego Fasoli, Olivier Faugeras, Stefano Panzeri

**Affiliations:** NeuroMathComp Laboratory, INRIA Sophia Antipolis Méditerranée, 2004 Route des Lucioles, BP 93, 06902 Valbonne, France; Neural Computation Laboratory, Center for Neuroscience and Cognitive Systems @Unitn, Istituto Italiano di Tecnologia, 38068 Rovereto, Italy

**Keywords:** Functional connectivity, Neural networks, Firing-rate network model, Perturbative theory, Stochastic systems, Graph theory

## Abstract

We introduce a new formalism for evaluating analytically the cross-correlation structure of a finite-size firing-rate network with recurrent connections. The analysis performs a first-order perturbative expansion of neural activity equations that include three different sources of randomness: the background noise of the membrane potentials, their initial conditions, and the distribution of the recurrent synaptic weights. This allows the analytical quantification of the relationship between anatomical and functional connectivity, i.e. of how the synaptic connections determine the statistical dependencies at any order among different neurons. The technique we develop is general, but for simplicity and clarity we demonstrate its efficacy by applying it to the case of synaptic connections described by regular graphs. The analytical equations so obtained reveal previously unknown behaviors of recurrent firing-rate networks, especially on how correlations are modified by the external input, by the finite size of the network, by the density of the anatomical connections and by correlation in sources of randomness. In particular, we show that a strong input can make the neurons almost independent, suggesting that functional connectivity does not depend only on the static anatomical connectivity, but also on the external inputs. Moreover we prove that in general it is not possible to find a mean-field description à la Sznitman of the network, if the anatomical connections are too sparse or our three sources of variability are correlated. To conclude, we show a very counterintuitive phenomenon, which we call *stochastic synchronization*, through which neurons become almost perfectly correlated even if the sources of randomness are independent. Due to its ability to quantify how activity of individual neurons and the correlation among them depends upon external inputs, the formalism introduced here can serve as a basis for exploring analytically the computational capability of population codes expressed by recurrent neural networks.

**Electronic Supplementary Material:**

The online version of this article (doi:10.1186/s13408-015-0020-y) contains supplementary material 1.

## Introduction

The brain is a complex system whose information processing capabilities critically rely on the interactions between neurons. One key factor that determines interaction among neurons is the pattern of their *anatomical or structural connectivity*, namely the specification of all the synaptic wirings that are physically present between neurons. However, communication among neurons appears to change dynamically [1], suggesting the presence of not-yet understood network mechanisms that modulate the effective strength of a given connection. Understanding how the functional connectivity of a neural network (i.e. the set of statistical dependencies among different neurons or neural populations [2]) depends upon the anatomical connectivity and is further modulated by other network parameters has thus become a central problem in systems neuroscience [3–8].

In this article we introduce a new formalism for evaluating analytically the structure of dependencies among neurons in the finite-size firing-rate network with recurrent connections introduced in [9]. Although these dependencies are computed from neural activity in a number of ways [10], in most cases functional connectivity is inferred from computing the correlation among neurons or populations of neurons [2]. In this article, we therefore concentrate on computing the correlations among neurons in a firing-rate network, although we also discuss how to compute, with the same formalism, also other measures of functional connectivity (Sect. 5).

To our knowledge, the problem of determining analytically the correlation structure of a neural network has been begun to be investigated systematically only recently. This is in part due to the new experimental insights into functional connectivity among cortical neurons [3–8], and in part due to the focus on many previous mathematical studies of neural networks on the mean-field approximation. This approximation exploits the fact that (under certain hypotheses) neurons become independent in the thermodynamic limit when the number of neurons *N* in the network goes to infinity. This kind of mean-field approximation has been developed by Sznitman [11–13], Tanaka [14–16], McKean [17, 18] and others. According to it, if the neurons are independent at time $t=0$ (initial chaos), then in the thermodynamic limit this independence propagates to every $t>0$.^1^ This phenomenon of propagation of chaos has been studied in different kinds of neural network models [19–22]. However, recent studies have begun to investigate the more interesting and rich structure of correlations arising in descriptions of networks dynamics beyond the thermodynamic limit. For example, new studies considered finite-size networks with excitatory and inhibitory populations, where the firing rates are determined by a linear response theory [23–25]. These studies included in the network sources of Poisson randomness in the spike times [23, 24], as well as randomness originating from normal white noise in the background for the membrane potentials [25]. Pioneer approaches [26] relied on estimating correlation by using a perturbative expansion around the thermodynamic limit in the inverse number of neurons in the network. The method was developed for binary neurons, where the sources of randomness were the transitions between the two states of each neuron and the topology of the synaptic connections, and a similar model was reintroduced recently in [27] for large networks. In [28] the author considered an alternative way to calculate correlations as a function of the inverse number of neurons (which is known as the *linear noise approximation*) and applied it to homogeneous populations of identical neurons with random fluctuations in the firing-rates. In [29] the authors introduced a density functional approach adapted from plasma physics to study correlations in large systems, and applied it to a heterogeneous network of phase neurons with random initial conditions. Another effective approach is represented by large deviations theory. In [30–32] the authors considered a discrete-time network of rate neurons, whose sources of randomness were background Brownian motions for the membrane potentials and normally distributed synaptic weights.

Building on these previous attempts to study network correlations including finite-size effects that go beyond the mean-field approximation, here we develop an approach based upon a first-order perturbative expansion of the neural equations. We introduce randomness through three different sources: the background noise of the membrane potentials, their initial conditions and the distribution of the recurrent synaptic weights. These sources of variability are normally distributed and can be correlated, and their standard deviations are used as perturbative parameters. Using this formalism and this model, we quantify analytically how synaptic connections determine statistical dependencies at any order (not only at the pairwise level, as in previous studies) among different neurons. The technique developed in this article is general, but for simplicity we demonstrate its efficacy by applying it to the case of synaptic connections described by regular graphs. A regular graph is a graph in which each vertex has the same number of neighbors, so this means that we consider networks where each neuron receives and makes the same number of connections. While this assumption is of course biologically implausible, it is sufficient to show interesting and non-trivial behaviors and will be relaxed to study more plausible connections in our future studies. We use this formalism to investigate in detail how the correlation structure depends on the strength of the external input to the network. We find that external input exerts profound and sometimes counterintuitive changes in the correlation among neurons: for example, a strong input can make the neurons almost independent. Moreover, we prove that in general it is not possible to find a mean-field description à la Sznitman of the neural network, due to the absence of chaos, if the anatomical connections are too sparse or our three sources of variability are correlated. This demonstrates the fairly limited range of applicability of the mean-field approximation. Finally, we also show a very counterintuitive phenomenon, which we call *stochastic synchronization*, through which neurons become almost perfectly correlated even if the sources of randomness are independent.

This article is organized as follows. In Sect. 2 we describe the details of the firing-rate network we use. We then develop a first-order perturbative expansion (Sect. 3) that allows the approximate analytical calculation, for a generic anatomical connectivity matrix, of the membrane potentials and the firing rates of the network. (In this section we assume the reader to be familiar with stochastic calculus [33, 34].) Then we use this formula for the membrane potentials and the firing rates in Sect. 4 to calculate analytically the pairwise and higher-order correlation structure of the network and the joint probability distribution for both the membrane potentials and the firing rates. In Sect. 5 we briefly discuss how other measures of functional connectivity can be evaluated analytically through our theory. In Sect. 6 we specialize to the case of regular graphs and we investigate network dynamics using some explicit examples of anatomical connectivity. We start by considering relatively simple cases, in particular a block-circulant graph with circulant blocks (Sect. 6.1) and a more general case of symmetric undirected graphs (Sect. 6.2). Then in Sect. 6.3 we conclude by showing how to extend the theory to highly complex regular graphs and by discussing also some possible extensions to irregular networks. In Sect. 7 we investigate the goodness of our perturbative approach by comparing it to the numerical simulation of the network’s equations. In Sect. 8 we show that the correlation structure depends dynamically on the external input of the network. In Sect. 9 we demonstrate with counterexamples that in general Sznitman’s mean-field approximation cannot be applied to the network in the case when the sources of randomness are correlated (Sect. 9.1) or when the anatomical connectivity matrix is too sparse (Sect. 9.2). In Sect. 10 we introduce the phenomenon of stochastic synchronization. Finally, in Sect. 11 we discuss the implications of our results as well as the strengths and limitations of our mathematical approach.

## Description of the Model

**Fig. 1:**
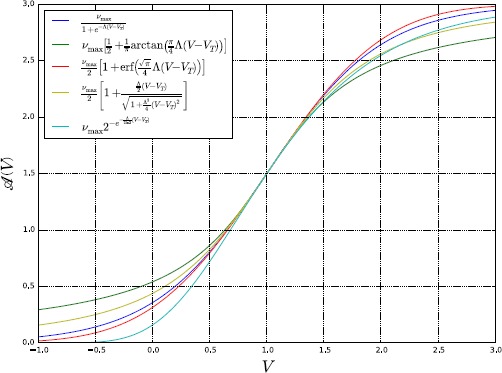
Plot of the sigmoidal activation functions (2.2) for $\nu_{\mathrm{max}}=3$, $\varLambda=2$, and $V_{T}=1$

We suppose that the neural network is described by the following firing-rate model [9]:

2.1 $$ \textstyle\begin{cases} dV_{i} (t )= [-\frac{1}{\tau}V_{i} (t )+\frac{1}{M_{i}}\sum_{j=0}^{N-1}J_{ij} (t )\mathscr {A} (V_{j} (t ) )+I_{i} (t ) ]\,dt \\ \hphantom{dV_{i} (t )={}}{}+\sigma_{0}\,d\mathscr{B}_{i} (t ), \\ V_{i} (0 )= \mu_{i}+\sigma_{1}\mathscr{N}_{i}, \end{cases} $$

with $i=0,\ldots,N-1$, where the total number of neurons *N* is finite. Here $V_{i} (t )$ is the membrane potential of the *i*th neuron, $I_{i} (t )$ is its external time-varying input current and *τ* is the membrane time constant describing the speed of convergence of the membrane potential to its resting state. Note that the external input can assume both positive and negative values, modeling the effect of prevailingly depolarizing or hyperpolarizing external influences, respectively. Moreover, $J_{ij} (t )$ is the synaptic weight from the *j*th to the *i*th neuron, while $M_{i}$ is the number of incoming connections of the *i*th neuron. In graph theory this quantity is called *incoming vertex degree* and its role will be explained later in this section. $\mathscr {A} (\cdot )$ represents a generic activation function, which converts the membrane potential *V* of a neuron into its corresponding firing rate $\nu =\mathscr{A} (V )$. A typical choice is to consider *S*-shaped (or sigmoidal) activation functions, because they are biologically plausible and their saturation for $\vert V\vert \rightarrow\infty$ ensures the boundedness of the solutions of Eq. (2.1). Some classic examples are shown below:

2.2 $$ \mathscr{A} (V )= \textstyle\begin{cases} \frac{\nu_{\mathrm{max}}}{1+e^{-\varLambda (V-V_{T} )}} & \mbox{(Logistic)}, \\ \nu_{\mathrm{max}} [\frac{1}{2}+\frac{1}{\pi}\arctan (\frac{\pi}{4}\varLambda (V-V_{T} ) ) ] & \mbox{(Inverse Tangent)}, \\ \frac{\nu_{\mathrm{max}}}{2} [1+\operatorname{erf} (\frac {\sqrt{\pi}}{4}\varLambda (V-V_{T} ) ) ] & \mbox{(Gauss Error)}, \\ \frac{\nu_{\mathrm{max}}}{2} [1+\frac{({\varLambda }/{2}) (V-V_{T} )}{\sqrt{1+({\varLambda^{2}}/{4}) (V-V_{T} )^{2}}} ] & \mbox{(Algebraic)}, \\ \nu_{\mathrm{max}}2^{-e^{-({\varLambda}/{(2\ln2)}) (V-V_{T} )}} & \mbox{(Gompertz)}, \\ \vdots \end{cases} $$

where in the above $\nu_{\mathrm{max}}$ is the maximum firing rate, *Λ* determines the speed with which the neuron switches from a low ($\nu\approx0$) to a high ($\nu\approx\nu _{\mathrm{max}}$) firing rate, and $V_{T}$ is the threshold between low and high firing rates, namely the value of the membrane potential such that $\nu=\frac {\nu_{\mathrm{max}}}{2}$. An example of the functions (2.2) is shown in Fig. 1 for some particular values of $\nu_{\mathrm{max}}$, *Λ*, and $V_{T}$.

The functions $\mathscr{B}_{i} (t )$, the first of the three sources of randomness introduced in the network, are non-fractional Brownian motions (or in other terms, Wiener processes with independent increments). They can be equivalently interpreted as a background noise for the membrane potentials $V_{i} (t )$ or as the stochastic component of the external input $I_{i} (t )$. $\sigma_{0}$ is the standard deviation (or intensity) of the noise, that for simplicity is supposed to be the same for all the neurons and constant in time. This is the first perturbative parameter that will be used in Sect. 3 to develop a first-order perturbative expansion of the neural equations. In general the Brownian motions are correlated according to the following covariance structure, chosen in order to keep the analysis as simple as possible:

2.3 $$ \begin{aligned} \operatorname{Cov} \biggl( \frac{d\mathscr{B}_{i} (t )}{dt},\frac {d\mathscr{B}_{j} (s )}{ds} \biggr) ={}&C_{ij}^{\mathscr {B}} \delta (t-s ), \\ C_{ij}^{\mathscr{B}} ={}& \textstyle\begin{cases} 1 & \mbox{if } i=j, \\ C^{ (0 )} & \mbox{otherwise}, \end{cases}\displaystyle \end{aligned} $$

or in other terms $\operatorname{Cov} (\frac{d\mathscr {B}_{i} (t )}{dt},\frac{d\mathscr{B}_{j} (s )}{ds} )= [\delta_{ij}+C^{ (0 )} (1-\delta_{ij} ) ]\delta (t-s )$. Here $\delta_{ij}$ is the Kronecker delta, $\delta (\cdot )$ is the Dirac delta function and $C^{ (0 )}$ represents the correlation between two different Brownian motions (the derivative of the Brownian motion with respect to time here is meant in the weak sense of distributions and is interpreted as white noise). The covariance matrix must be positive-semidefinite. Since it is symmetric, then it is positive-semidefinite if and only if its eigenvalues are non-negative. Moreover, with our choice, the covariance matrix is circulant, therefore its eigenvalues are $1+C^{ (0 )} (N-1 )$ and $1-C^{ (0 )}$, with algebraic multiplicity 1 and $N-1$, respectively. Therefore the matrix is positive-semidefinite if and only if $\frac{1}{1-N}\leq C^{ (0 )}\leq1$. Note that there are no technical obstructions to increasing the complexity of this correlation structure, if desired.

The initial conditions $V_{i} (0 )$ are normally distributed around their mean $\mu_{i}$ with standard deviation $\sigma_{1}$, the second of our perturbative parameters. The stochastic variables $\mathscr{N}_{i}$ (see Eq. (2.1)) are normally distributed with zero mean and covariance matrix:

2.4 $$ \operatorname{Cov} (\mathscr{N}_{i}, \mathscr{N}_{j} )= \textstyle\begin{cases} 1 & \mbox{if } i=j, \\ C^{ (1 )} & \mbox{otherwise}, \end{cases} $$

namely $\operatorname{Cov} (\mathscr{N}_{i},\mathscr {N}_{j} )=\delta_{ij}+C^{ (1 )} (1-\delta_{ij} )$. The coefficient $C^{ (1 )}$ is the correlation between pairs of membrane potentials at time $t=0$. As before, the covariance matrix must be positive-semidefinite, and this is true if and only if $\frac {1}{1-N}\leq C^{ (1 )}\leq1$. Again, it is possible to increase the complexity of this correlation structure, if desired.

The third and last source of randomness in the network is represented by the synaptic connectivity $J (t )$. We assume that each entry $J_{ij} (t )$ is normally distributed around its mean $\overline{J}_{ij} (t )$ with standard deviation $\sigma_{2}$ (the third perturbative parameter used in Sect. 3), or in other terms:

2.5 $$ J_{ij} (t )=T_{ij} \bigl( \overline{J}_{ij} (t )+\sigma_{2}W_{ij} \bigr), $$

$W_{ij}$ are zero mean normal stochastic variables (their covariance structure is shown below, see Eq. (2.6)), while the matrix *T* represents the *topology* of the connectivity matrix, namely the mere absence ($T_{ij}=0$) or presence ($T_{ij}=1$) of the synaptic connection from the *j*th neuron to the *i*th neuron, for all the pairs of neurons $(i,j )$. So if the connection is present, its strength is given by $\overline {J}_{ij} (t )+\sigma_{2}W_{ij}$, otherwise it is equal to zero. Below we show an example of connectivity matrix in a network of four neurons and its corresponding topology:

$$\begin{aligned} J (t )&=\left [ \textstyle\begin{array}{@{}c@{\quad}c@{\quad}c@{\quad}c@{}} 0 & 0 & 7.1+2\cos (2t ) & 3.6 \\ 2.3+\sin (5t ) & 0 & 10.3 & 0 \\ 0.9 & 1.1 & 0 & 4.8-\arctan (3t ) \\ 0 & 5.4- (1+t )^{-5} & 7.5+e^{-4t} & 0 \end{array}\displaystyle \right ], \\ T&= \left [ \textstyle\begin{array}{@{}c@{\quad}c@{\quad}c@{\quad}c@{}} 0 & 0 & 1 & 1 \\ 1 & 0 & 1 & 0 \\ 1 & 1 & 0 & 1 \\ 0 & 1 & 1 & 0 \end{array}\displaystyle \right ] . \end{aligned}$$

In graph theory, *T* is known as the *adjacency matrix* of the unweighted graph of the network, and in this article is supposed to be deterministic and time-independent. Therefore the only source of randomness in the synaptic matrix is represented by $W_{ij}$, whose covariance structure is chosen as follows:

2.6 $$ \operatorname{Cov} (W_{ij},W_{kl} )= \textstyle\begin{cases} 0 & \mbox{if } T_{ij}=0 \mbox{ and/or } T_{kl}=0, \\ 1 & \mbox{if } i=k \mbox{ and } j=l \mbox{ and } T_{ij}=1, \\ C^{ (2 )} & \mbox{otherwise}, \end{cases} $$

or in other terms $\operatorname{Cov} (W_{ij},W_{kl} )=T_{ij}T_{kl} [\delta_{ik}\delta_{jl}+C^{ (2 )} (1-\delta_{ik}\delta_{jl} ) ]$. This simply means that $W_{ij}$ has zero (unit) variance if the connection $i\leftarrow j$ is absent (present), while the covariance between $W_{ij}$ and $W_{kl}$ is zero ($C^{ (2 )}$) if at least one of the connections $i\leftarrow j$ and $k\leftarrow l$ is absent (they are both present). As for the covariance structures (2.3) and (2.4), also (2.6) can be made more complicated, if desired. With our choice, the range of allowed values of $C^{ (2 )}$ depends on the topology of the connectivity matrix. In order to find this range, we start by vectorizing the matrices *W* and *T* as follows:

$$\mathbf {W}\overset{\mathrm{def}}{=}\operatorname{vec} (W ), \qquad \mathbf {T}\overset{\mathrm{def}}{=}\operatorname {vec} (T ). $$

This allows us to reinterpret the $N\times N$ matrix-variate random variable *W* as a $N^{2}$-dimensional multivariate vector **W** with a $N^{2}\times N^{2}$ covariance matrix $\operatorname{Cov} (\mathbf {W}_{i},\mathbf {W}_{j} )$. Now we call *Z* the number of absent connections (i.e. the number of zeros in the matrix *T*), and we suppose that the vectorization is such that $\mathbf {T}_{i}=0$ for $i=0,\ldots,Z-1$. According to (2.6), if we call *Θ* the covariance matrix of **W**, we obtain

$$\varTheta=\left [ \textstyle\begin{array}{@{}c@{\quad}c@{}} \mathit{0}_{Z,Z} & \mathit{0}_{Z,N^{2}-Z} \\ \mathit{0}_{N^{2}-Z,Z} & \varOmega \end{array}\displaystyle \right ], \quad \varOmega=\left [ \textstyle\begin{array}{@{}c@{\quad}c@{\quad}c@{\quad}c@{}} 1 & C^{ (2 )} & \cdots& C^{ (2 )} \\ C^{ (2 )} & 1 & \cdots& C^{ (2 )} \\ \vdots& \vdots& \ddots& \vdots \\ C^{ (2 )} & C^{ (2 )} & \cdots& 1 \end{array}\displaystyle \right ] $$

where $\mathit{0}_{X,Y}$ is the $X\times Y$ null matrix. Since *Θ* is a $2\times2$ block matrix, its characteristic polynomial is

$$\begin{aligned} \det (\varTheta-\lambda \mathrm {Id}_{N^{2}} )&=\det (-\lambda \mathrm {Id}_{Z} )\det (\varOmega-\lambda \mathrm {Id}_{N^{2}-Z} ) \\ &= (-\lambda )^{Z} \bigl[1+C^{ (2 )} \bigl(N^{2}-Z-1 \bigr)-\lambda \bigr] \bigl(1-C^{ (2 )}-\lambda \bigr)^{N^{2}-Z-1} \end{aligned}$$

where $\mathrm {Id}_{K}$ is the $K\times K$ identity matrix. Therefore *Θ* has eigenvalues 0, $1+C^{ (2 )} (N^{2}-Z-1 )$ and $1-C^{ (2 )}$, with algebraic multiplicity *Z*, 1 and $N^{2}-Z-1$, respectively. This means that *Θ* is a true covariance matrix if and only if $\frac{1}{1+Z-N^{2}}\leq C^{ (2 )}\leq1$, where *Z* depends on the topology of the network.

In order to avoid biologically unrealistic sign changes of the synaptic weights, $\overline{J}_{ij} (t )$ should not change sign during time evolution. Moreover, $\vert \overline{J}_{ij} (t )\vert $ must be much larger than $\sigma_{2}$ for every *i*, *j*, and *t*, because in this way the probability that $\overline{J}_{ij} (t )+\sigma_{2}W_{ij}$ changes sign from trial to trial is small:

$$\begin{aligned} &\mathrm{P} \bigl(\operatorname{sgn} \bigl(\overline{J}_{ij} (t )+ \sigma_{2}W_{ij} \bigr)\neq\operatorname{sgn} \bigl( \overline {J}_{ij} (t ) \bigr) \bigr) \\ &\quad=\frac{1}{2} \biggl[1- \operatorname{erf} \biggl(\frac{\vert \overline{J}_{ij} (t )\vert }{\sqrt{2}\sigma_{2}} \biggr) \biggr]\approx \frac{1}{2\sqrt{\pi }}\frac{\sigma_{2}}{\vert \overline{J}_{ij} (t )\vert }e^{- ({\overline{J}_{ij} (t )}/{\sigma _{2}} )^{2}} \end{aligned}$$

having used an asymptotic expansion of the error function for $\frac{\vert \overline{J}_{ij} (t )\vert }{\sigma _{2}}\gg1$.

Since in Sect. 3 we will solve perturbatively the system of equations (2.1), it is clear that this cannot be accomplished with the current formulation of the synaptic weights. Actually, even if our differential equations were linear, in general it would not be possible to solve them exactly, since their coefficients are time dependent, due to $\overline {J}_{ij} (t )$. Linear differential equations with variable coefficients can be solved perturbatively in terms of a *Magnus expansion* [35], but this is not the approach followed in our work. In order to unify the perturbative expansion introduced in this article with the problem of the variation of the coefficients, we rewrite the matrix $\overline {J}_{ij} (t )$ as follows:

2.7 $$ \overline{J}_{ij} (t )=\overline{J}_{ij}^{c}+ \sigma _{3}\overline{J}_{ij}^{v} (t ) $$

where $\overline{J}_{ij}^{c}$ is constant in time, while $\overline{J}_{ij}^{v} (t )$ is variable. $\sigma_{3}$ is assumed to be small and will be used as a perturbative parameter in Sect. 3. In this way the time variability of the synaptic matrix is treated perturbatively, as for the three sources of randomness in the network, and this will allow us to find analytical solutions for the perturbative expansion developed in the next section. Moreover, the variable part of the synaptic weights should satisfy the constraint $\vert \overline{J}_{ij}^{v} (t )\vert \in [0,1 ]$ for all *t*, because according to (2.7) this implies $\max_{t}\vert \overline{J}_{ij} (t )-\overline{J}_{ij}^{c}\vert \leq\sigma_{3}$. In this way we ensure that at every time instant $\overline {J}_{ij} (t )$ is not too different from $\overline{J}_{ij}^{c}$, and therefore that a first-order perturbative expansion of the neural equations provides a good approximation of the real dynamics of the network.

It is also important to observe that when a neuron receives more and more connections from the other neurons (i.e. when $M_{i}\rightarrow \infty$, see Eq. (2.1)), the sum $\sum_{j=0}^{N-1}J_{ij} (t )\mathscr{A} (V_{j} (t ) )$ in (2.1) is divergent, therefore the limit of large and densely connected networks would not be well defined. This explains the need to introduce the factor $\frac{1}{M_{i}}$ to normalize the divergent sum. For later purpose, it is useful to express $M_{i}$ in terms of the topology of the network:

2.8 $$ M_{i}=\sum_{j=0}^{N-1}T_{ij} . $$

Finally, as we did for $\overline{J}_{ij} (t )$, we suppose that the external input current is deterministic (if we interpret $\mathscr{B}_{i} (t )$ as the background noise of the membrane potentials) and given by

2.9 $$ I_{i} (t )=I_{i}^{c}+ \sigma_{4}I_{i}^{v} (t ) $$

where $I_{i}^{c}$ is constant in time, while $I_{i}^{v} (t )$ is variable. $\sigma_{4}$ is our last perturbative parameter, and together with $\sigma_{3}$ quantifies the time variability of the network. As for the synaptic weights, we have the constraint $\vert I_{i}^{v} (t )\vert \in [0,1 ]$, because according to (2.9) this implies $\max_{t}\vert I_{i} (t )-I_{i}^{c}\vert \leq\sigma_{4}$.

For simplicity, the three sources of randomness are supposed to be independent from each other, namely:

2.10 $$ \operatorname{Cov} \bigl(\mathscr{B}_{i} (t ),\mathscr {N}_{j} \bigr)=\operatorname{Cov} \bigl(\mathscr{B}_{i} (t ),W_{kl} \bigr)=\operatorname{Cov} (\mathscr {N}_{j},W_{kl} )=0 . $$

This assumption reduces considerably the complexity of the formula for the correlation structure that we will calculate in Sect. 4, but can be relaxed if desired.

This concludes our description of the neural equations, so now we have all the ingredients to develop a perturbative expansion of the system. This method is introduced in the next section, and will allow us to find a series of new results for the behavior of our stochastic neural network.

## Perturbative Expansion

As we said in the previous section, we want to develop a first-order perturbative expansion of the neural network in terms of the perturbative parameters $\sigma_{0}$–$\sigma_{4}$. To this purpose we define the following first-order expansion of the membrane potentials:

3.1 $$ V_{i} (t )\approx\mu_{i}+\sum _{m=0}^{4}\sigma _{m}Y_{i}^{ (m )} (t ) . $$

This ansatz will be used to obtain an approximate analytical solution of the system (2.1). The terms $\mu_{i}$ are the mean initial conditions of the membrane potentials (see Eq. (2.1)), while the functions $Y_{i}^{ (m )}$ have to be determined (here the superscript does not mean differentiation). Intuitively, the terms $\sigma _{m}Y_{i}^{ (m )}$ quantify the variation of the membrane potentials from the stationary solution $V_{i}=\mu_{i}$, due to the three sources of randomness and the time variability of the synaptic weights and the external input currents.

The functions $Y_{i}^{ (m )}$ can be determined by substituting the perturbative expansion (3.1) and the expressions (2.5) + (2.7) and (2.9) for, respectively, the synaptic weights and the external input current, into the system (2.1). If all the parameters $\sigma_{m}$ are small enough, we can expand the activation function $\mathscr{A} (V_{i} )$ in a Taylor series about $\mu_{i}$. In order to be rigorous, we have to determine the radius of convergence $r (\mu_{i} )$ of the Taylor expansion for every value of $\mu_{i}$ and to check if the radius is big enough compared to $\sigma_{m}$, because otherwise the series does not converge. Actually, the various $\sigma_{m}$ determine the order of magnitude of the fluctuations of $V_{i}$ around $\mu_{i}$, therefore it is important to check if $V_{i}$ lies inside the interval of convergence of the Taylor expansion (this will be quantified more rigorously at the end of Sect. 4). In Appendix A we evaluate $r (\mu_{i} )$ for two examples of $\mathscr {A} (V_{i} )$, namely the logistic and the inverse tangent activation functions (see (2.2)). In both cases we obtain that the radius decreases with the slope parameter *Λ*, and since all the sigmoidal functions are qualitatively similar, it is reasonable to assume that this result holds for all of them. Therefore, supposing that *Λ* is small enough, the Taylor expansion of $\mathscr{A} (V_{i} )$ truncated at the first order is

3.2 $$ \mathscr{A} \Biggl(\mu_{i}+\sum _{m=0}^{4}\sigma_{m}Y_{i}^{ (m )} (t ) \Biggr)\approx\mathscr{A} (\mu _{i} )+\mathscr{A}' (\mu_{i} )\sum_{m=0}^{4}\sigma _{m}Y_{i}^{ (m )} (t ) . $$

Now we substitute this expansion inside the neural equations (2.1) and we equate the terms with the same *σ* coefficients, obtaining

3.3 $$\begin{aligned} \mu_{i} =& \tau \Biggl[\frac {1}{M_{i}}\sum _{j=0}^{N-1}T_{ij}\overline{J}_{ij}^{c} \mathscr{A} (\mu _{j} )+I_{i}^{c} \Biggr], \end{aligned}$$

3.4 $$\begin{aligned} dY_{i}^{ (0 )} (t ) =& \Biggl[\sum _{j=0}^{N-1}\mathcal{J}_{ij}Y_{j}^{ (0 )} (t ) \Biggr]\,dt+d\mathscr{B}_{i} (t ), \end{aligned}$$

3.5 $$\begin{aligned} dY_{i}^{ (1 )} (t ) =& \Biggl[\sum _{j=0}^{N-1}\mathcal{J}_{ij}Y_{j}^{ (1 )} (t ) \Biggr]\,dt, \end{aligned}$$

3.6 $$\begin{aligned} dY_{i}^{ (2 )} (t ) =& \Biggl[\sum _{j=0}^{N-1}\mathcal{J}_{ij}Y_{j}^{ (2 )} (t )+\frac{1}{M_{i}}\sum_{j=0}^{N-1}T_{ij}W_{ij} \mathscr{A} (\mu _{j} ) \Biggr]\,dt, \end{aligned}$$

3.7 $$\begin{aligned} dY_{i}^{ (3 )} (t ) =& \Biggl[\sum _{j=0}^{N-1}\mathcal{J}_{ij}Y_{j}^{ (3 )} (t )+\frac{1}{M_{i}}\sum_{j=0}^{N-1}T_{ij} \overline{J}_{ij}^{v} (t )\mathscr{A} (\mu_{j} ) \Biggr]\,dt, \end{aligned}$$

3.8 $$\begin{aligned} dY_{i}^{ (4 )} (t ) =& \Biggl[\sum _{j=0}^{N-1}\mathcal{J}_{ij}Y_{j}^{ (4 )} (t )+I_{i}^{v} (t ) \Biggr]\,dt \end{aligned}$$

where

3.9 $$\begin{aligned} \mathcal{J}_{ij} =& \textstyle\begin{cases} -\frac{1}{\tau} & \mbox{if } i=j, \\ \frac{1}{M_{i}}J_{ij}^{\mathrm{eff}} & \mbox{otherwise}, \end{cases}\displaystyle \end{aligned}$$

3.10 $$\begin{aligned} J_{ij}^{\mathrm{eff}} \overset{\mathrm{def}}{=}& T_{ij}\overline {J}_{ij}^{c} \mathscr{A}' (\mu_{j} ). \end{aligned}$$

$\mathcal{J}$ is the Jacobian matrix of the network, while $J^{\mathrm{eff}}$ can be interpreted as the real anatomical connectivity matrix that the system would have if it were linear. For this reason we call it the *effective connectivity matrix* of the network, and it should not be confused with the effective connectivity discussed in [2].

Now we observe that equations (3.3) are algebraic and non-linear, therefore in general they must be solved numerically. Eventually, it is possible to obtain analytical solutions when the activation function is approximated by a piecewise linear function. Moreover, (3.4) ((3.5)–(3.8)) are linear stochastic (ordinary) differential equations with constant coefficients, therefore can be solved analytically as a function of $\mu_{i}$, which are supposed to be known from the solution of (3.3). The *fundamental matrix*$\varPhi (t )$ of the system is

$$\varPhi (t )=e^{\mathcal{J}t} $$

where $\mathcal{J}$ is given by (3.9). In this article we consider only cases when $\mathcal{J}$ is diagonalizable, so we can calculate the matrix exponential as follows:

3.11 $$ e^{\mathcal{J}t}=PD (t )P^{-1} $$

where $D (t )=\operatorname{diag} (e^{\widetilde {\lambda }_{0}t},\ldots,e^{\widetilde{\lambda}_{N-1}t} )$ and $\widetilde{\lambda}_{i}$ are the eigenvalues of $\mathcal{J}$, while *P* is an $N\times N$ matrix whose columns are composed of the eigenvectors of $\mathcal{J}$. The differential equations (3.4)–(3.8) are linear with constant coefficients since, as explained in the previous section, we have used the perturbative approach to fix the problem of the time variability of the coefficients. So their solutions are obtained straightforwardly as follows:

3.12 $$\begin{aligned} Y_{i}^{ (0 )} (t ) =& \sum _{j=0}^{N-1}\int_{0}^{t} \varPhi_{ij} (t-s )\,d\mathscr{B}_{j} (s ), \end{aligned}$$

3.13 $$\begin{aligned} Y_{i}^{ (1 )} (t ) =& \sum _{j=0}^{N-1}\varPhi _{ij} (t ) \mathscr{N}_{j}, \end{aligned}$$

3.14 $$\begin{aligned} Y_{i}^{ (2 )} (t ) =& \sum _{j,k=0}^{N-1}\frac {1}{M_{j}}T_{jk}W_{jk} \mathscr{A} (\mu_{k} )\int_{0}^{t} \varPhi_{ij} (t-s )\,ds, \end{aligned}$$

3.15 $$\begin{aligned} Y_{i}^{ (3 )} (t ) =& \sum _{j,k=0}^{N-1}\frac {1}{M_{j}}T_{jk} \mathscr{A} (\mu_{k} )\int_{0}^{t}\varPhi _{ij} (t-s )\overline{J}_{jk}^{v} (s )\,ds, \end{aligned}$$

3.16 $$\begin{aligned} Y_{i}^{ (4 )} (t ) =& \sum _{j=0}^{N-1}\int_{0}^{t} \varPhi_{ij} (t-s )I_{j}^{v} (s )\,ds. \end{aligned}$$

By substituting the solutions (3.12)–(3.16) inside (3.1), we obtain an approximate formula for the membrane potentials of the neural network. Moreover, since $\nu=\mathscr{A} (V )$, (3.2) provides a perturbative expression for the firing rates.

Now with these results we can determine analytically the behavior of the neural network, starting from its correlation structure and probability density, which are discussed in the next section.

## Cross-Correlation and Probability Density

The aim of this section is to compute the statistical dependencies among the activity of different neurons.

We first calculate the Pearson cross-correlation among pairs of neurons, which is the simplest and most commonly used measure of functional connectivity [10]. This is defined as follows:

$$\operatorname{Corr}_{2} \bigl(V_{i} (t ),V_{j} (t ) \bigr)=\frac{\operatorname{Cov}_{2} (V_{i} (t ),V_{j} (t ) )}{\sqrt{\operatorname{Var} (V_{i} (t ) )\operatorname{Var} (V_{j} (t ) )}} . $$

The subscript “2” has been introduced to stress the fact that it is a pairwise correlation between two neurons. We note that the above expression quantifies time-dependent instantaneous correlations at any given time *t*. This equation can easily be extended to higher-order correlations between triplets, quadruplets, etc. of neurons. The most straightforward generalization of the pairwise covariance to the case of *n* neurons would be

$$\operatorname{Cov}_{n} \bigl(V_{i_{0}} (t ),\ldots ,V_{i_{n-1}} (t ) \bigr)=\overline{\prod_{j=0}^{n-1} \bigl(V_{i_{j}} (t )-\overline{V}_{i_{j}} (t ) \bigr)} $$

where the bar represents the statistical mean over trials computed at time *t*. This is the multivariate moment $M_{1,\ldots,1}$ of the functions $V_{i_{0}} (t ),\ldots,V_{i_{n-1}} (t )$ about the mean $(\overline{V}_{i_{0}} (t ),\ldots ,\overline{V}_{i_{n-1}} (t ) )$. However, this measure is not yet normalized to lie in the range $[-1,1 ]$. To achieve this purpose, we observe that

$$\Biggl\vert \overline{\prod_{j=0}^{n-1} \bigl(V_{i_{j}} (t )-\overline{V}_{i_{j}} (t ) \bigr)}\Biggr\vert \leq\overline {\Biggl\vert \prod_{j=0}^{n-1} \bigl(V_{i_{j}} (t )-\overline {V}_{i_{j}} (t ) \bigr)\Biggr\vert }\leq \Biggl[\prod_{j=0}^{n-1} \overline{\bigl\vert V_{i_{j}} (t )-\overline {V}_{i_{j}} (t ) \bigr\vert ^{n}} \Biggr]^{{1}/{n}} $$

having used the fact that $\vert x+y\vert \leq \vert x\vert +\vert y\vert $ at the first step and a special case of the Hölder inequality at the second. This means that the function

4.1 $$ \operatorname{Corr}_{n} \bigl(V_{i_{0}} (t ), \ldots ,V_{i_{n-1}} (t ) \bigr)\overset{\mathrm{def}}{=}\frac {\overline{ \prod_{j=0}^{n-1} (V_{i_{j}} (t )-\overline{V}_{i_{j}} (t ) )}}{\sqrt[n]{ \prod_{j=0}^{n-1} \overline{\vert V_{i_{j}} (t )-\overline {V}_{i_{j}} (t )\vert ^{n}}}} $$

is such that $\vert \operatorname{Corr}_{n} (V_{i_{0}} (t ),\ldots,V_{i_{n-1}} (t ) )\vert \leq1$. Therefore, we will use Eq. (4.1) to quantify correlations at any order. Note that Eq. (4.1) is equivalent for $n=2$ to the pairwise Pearson coefficient, and thus Eq. (4.1) includes also the pairwise correlation as a special case.

From (3.12)–(3.14) and remembering that $\overline{\mathscr{B}}_{i} (t )=\overline{\mathscr{N}}_{i}=\overline{W}_{ij}=0$, we obtain $\overline{Y}_{i}^{ (0 )} (t )=\overline{Y}_{i}^{ (1 )} (t )=\overline {Y}_{i}^{ (2 )} (t )=0$ therefore, by using (3.1), we have

4.2 $$ \mathfrak{N}_{i} (t )\overset{\mathrm{def}}{=}V_{i} (t )-\overline{V}_{i} (t )=\sum _{m=0}^{2}\sigma _{m}Y_{i}^{ (m )} (t ) . $$

Clearly $(\mathfrak{N}_{i_{0}} (t ),\ldots,\mathfrak {N}_{i_{n-1}} (t ) )$ is a zero mean multivariate normal process, whose covariance matrix is given by

4.3 $$ \operatorname{Cov}_{2} \bigl(\mathfrak{N}_{i} (t ),\mathfrak {N}_{j} (t ) \bigr)=\overline{\mathfrak{N}_{i} (t )\mathfrak{N}_{j} (t )}= \sum_{m=0}^{2} \sigma _{m}^{2}\overline{Y_{i}^{ (m )} (t )Y_{j}^{ (m )} (t )} $$

due to (2.10). The terms $\overline {Y_{i}^{ (m )} (t )Y_{j}^{ (m )} (t )}$ are calculated from the relations (2.3), (2.4) and (2.6):

4.4 $$\begin{aligned} &\begin{aligned}[b]\overline{Y_{i}^{ (0 )} (t )Y_{j}^{ (0 )} (t )}={}& \bigl(1-C^{ (0 )} \bigr)\sum_{k=0}^{N-1} \int_{0}^{t}\varPhi_{ik} (t-s )\varPhi _{jk} (t-s )\,ds \\ &{} +C^{ (0 )} \sum_{k,l=0}^{N-1} \int _{0}^{t}\varPhi _{ik} (t-s ) \varPhi_{jl} (t-s )\,ds, \end{aligned} \end{aligned}$$

4.5 $$\begin{aligned} &\begin{aligned}[b]\overline{Y_{i}^{ (1 )} (t )Y_{j}^{ (1 )} (t )}={}& \bigl(1-C^{ (1 )} \bigr)\sum_{k=0}^{N-1} \varPhi_{ik} (t )\varPhi_{jk} (t ) \\ &{} +C^{ (1 )} \sum_{k,l=0}^{N-1} \varPhi_{ik} (t )\varPhi_{jl} (t ), \end{aligned} \end{aligned}$$

4.6 $$\begin{aligned} &\begin{aligned}[b] &\overline{Y_{i}^{ (2 )} (t )Y_{j}^{ (2 )} (t )} \\ &\quad= \bigl(1-C^{ (2 )} \bigr)\sum_{k=0}^{N-1} \frac{\chi_{k}}{M_{k}^{2}} \biggl[\int_{0}^{t}\varPhi _{ik} (t-s )\,ds \biggr] \biggl[\int_{0}^{t} \varPhi_{jk} (t-s )\,ds \biggr] \\ &\qquad{} +C^{ (2 )} \sum_{k,l=0}^{N-1} \frac{\psi_{k}\psi _{l}}{M_{k}M_{l}} \biggl[\int_{0}^{t} \varPhi_{ik} (t-s )\,ds \biggr] \biggl[\int_{0}^{t} \varPhi_{jl} (t-s )\,ds \biggr] \end{aligned} \end{aligned}$$

where

4.7 $$ \begin{aligned} \chi_{i}\overset{ \mathrm{def}}{=} {} & \sum_{j=0}^{N-1}T_{ij}^{2} \mathscr{A}^{2} (\mu_{j} ), \\ \psi_{i}\overset{\mathrm{def}}{=} {} & \sum _{j=0}^{N-1} T_{ij}\mathscr{A} ( \mu_{j} ). \end{aligned} $$

Using the Isserlis theorem [36], and noting that we assumed that our sources of randomness are normally distributed, we obtain that the numerator of (4.1) is equal to zero when *n* is odd (in general this is false for non-normal processes), otherwise

4.8 $$ \overline{ \prod_{j=0}^{n-1} \bigl(V_{i_{j}} (t )-\overline{V}_{i_{j}} (t ) \bigr)}=\sum \prod\overline {\mathfrak{N}_{i_{j}} (t ) \mathfrak{N}_{i_{k}} (t )} $$

where ∑∏ means summing over all the distinct $\frac {n!}{2^{{n}/{2}} ({n}/{2} )!}$ ways of partitioning $\mathfrak{N}_{i_{0}} (t ),\ldots ,\mathfrak{N}_{i_{n-1}} (t )$ into pairs. This completes the calculation of the numerator of (4.1).

For the denominator we use the formula of the absolute moments of a normal process, therefore for *n* even we obtain

4.9 $$ \sqrt[n]{ \prod_{j=0}^{n-1} \overline{\bigl\vert \mathfrak {N}_{i_{j}} (t )\bigr\vert ^{n}}}=\frac{n!}{2^{{n}/{2}} ({n}/{2} )!} \prod_{j=0}^{n-1} \sqrt {\overline{\mathfrak{N}_{i_{j}}^{2}} (t )} $$

where $\overline{\mathfrak{N}_{i}^{2}} (t )$ is given by (4.3) and (4.4)–(4.6) for $i=j$. Finally, by substituting all these results into the definition (4.1), we obtain the final formula for the higher-order correlation of the network.

We observe that computation of Eq. (4.8) leads to a combinatorial problem, whose complexity is related to *n* and to the structure of the effective connectivity matrix $J^{\mathrm{eff}}$. To simplify matters, in Appendix B we will calculate the higher-order correlation for a generic *n* in the case of a complete graph (i.e. a fully connected network).

We also observe that in the special case $n=2$ our formula reduces simply to

4.10 $$ \operatorname{Corr}_{2} \bigl(V_{i} (t ),V_{j} (t ) \bigr)=\frac{ \sum_{m=0}^{2} \sigma_{m}^{2}\overline {Y_{i}^{ (m )} (t )Y_{j}^{ (m )} (t )}}{\sqrt{ [ \sum_{m=0}^{2} \sigma_{m}^{2}\overline { [Y_{i}^{ (m )} (t ) ]^{2}} ] [ \sum_{m=0}^{2} \sigma_{m}^{2}\overline{ [Y_{j}^{ (m )} (t ) ]^{2}} ]}} $$

where again the terms $\overline{Y_{i}^{ (m )} (t )Y_{j}^{ (m )} (t )}$ and $\overline{ [Y_{i (j )}^{ (m )} (t ) ]^{2}}$ are given by (4.4)–(4.6), so in this case the combinatorial problem is absent. Due to its simplicity and in order to keep the article as short as possible, in the next sections we will evaluate explicitly only the pairwise correlation structure through (4.10) (therefore the subscript “2” in the notation $\operatorname{Corr}_{2} (\cdot,\cdot )$ will be omitted). The interested reader could apply the general Eq. (4.1) for the calculation of the correlation structure when $n>2$.

Neuroscientists make use of measures of correlation between firing rates, rather than between membrane potentials, to study cross-neuron communication. This is because only spiking events (and not subthreshold membrane fluctuations) are transmitted to other neurons. For this reason we also derive a formula for the correlation of the firing rates *ν*. Since in this model $\nu_{i}=\mathscr{A} (V_{i} )$, from (3.2) and (4.2) it is easy to prove that $\nu_{i} (t )-\overline{\nu }_{i} (t )=\mathscr{A}' (\mu_{i} )\mathfrak {N}_{i} (t )$. Therefore we have

$$\begin{aligned} \overline{ \prod_{j=0}^{n-1} \bigl( \nu_{i_{j}} (t )-\overline{\nu}_{i_{j}} (t ) \bigr)} =& \varsigma \overline{ \prod_{j=0}^{n-1} \bigl(V_{i_{j}} (t )-\overline{V}_{i_{j}} (t ) \bigr)}, \\ \sqrt[n]{ \prod_{j=0}^{n-1} \overline{\bigl\vert \nu_{i_{j}} (t )-\overline{\nu}_{i_{j}} (t )\bigr\vert ^{n}}} =& \varsigma\sqrt[n]{ \prod_{j=0}^{n-1} \overline{\bigl\vert V_{i_{j}} (t )-\overline{V}_{i_{j}} (t ) \bigr\vert ^{n}}}, \\ \varsigma =& \prod_{j=0}^{n-1} \mathscr{A}' (\mu _{i_{j}} ), \end{aligned}$$

having used the fact that $\mathscr{A}' (\mu_{i_{j}} )$ is always positive. This proves that

$$\operatorname{Corr}_{n} \bigl(\nu_{i_{0}} (t ),\ldots,\nu _{i_{n-1}} (t ) \bigr)=\operatorname{Corr}_{n} \bigl(V_{i_{0}} (t ),\ldots,V_{i_{n-1}} (t ) \bigr). $$

However, it is important to observe that the correlation structures of the firing rates and the membrane potentials are equivalent only at the first perturbative order, namely when all the parameters $\sigma_{m}$ are relatively small. At higher orders this equivalence does not hold anymore.

Now we have all the ingredients required to evaluate the joint probability distribution of the neural network. Since we have linearized the differential equations (2.1), at the first perturbative order the joint probability density of the system is a multivariate normal distribution. Denoting by ′ the matrix transposition operator and defining $\mathbf {V}\overset{\mathrm{def}}{=} (V_{0},\ldots,V_{N-1} )$, we obtain

4.11 $$\begin{aligned} p (\mathbf {V},t ) & =\frac{1}{\sqrt{ (2\pi )^{N}\vert \varSigma^{V} (t )\vert }}e^{-({1}/{2}) (\mathbf {V}-\overline{\mathbf {V}} (t ) )' (\varSigma^{V} (t ) )^{-1} (\mathbf {V}-\overline{\mathbf {V}} (t ) )}, \\ \overline{V}_{i} (t ) & =\mu_{i}+\sum _{m=3}^{4}\sigma _{m}Y_{i}^{ (m )} (t ), \\ \varSigma_{ij}^{V} (t ) & = \sum _{m=0}^{2} \sigma _{m}^{2} \overline{Y_{i}^{ (m )} (t )Y_{j}^{ (m )} (t )}. \end{aligned}$$

In a similar way, if we define $\boldsymbol{\nu}\overset{\mathrm {def}}{=} (\nu_{0},\ldots,\nu_{N-1} )$ and we remember that $\nu_{i}=\mathscr{A} (V_{i} )$, from (3.2) we obtain

4.12 $$\begin{aligned} p (\boldsymbol{\nu},t ) & =\frac{1}{\sqrt{ (2\pi )^{N}\vert \varSigma^{\nu} (t )\vert }}e^{-({1}/{2}) (\boldsymbol{\nu}-\overline{\boldsymbol{\nu}} (t ) )' (\varSigma^{\nu} (t ) )^{-1} (\boldsymbol{\nu}-\overline{\boldsymbol{\nu}} (t ) )}, \\ \overline{\nu}_{i} (t ) & =\mathscr{A} (\mu _{i} )+ \mathscr{A}' (\mu_{i} )\sum_{m=3}^{4} \sigma _{m}Y_{i}^{ (m )} (t ), \\ \varSigma_{ij}^{\nu} (t ) & =\mathscr{A}' (\mu _{i} )\mathscr{A}' (\mu_{j} ) \sum _{m=0}^{2} \sigma_{m}^{2} \overline{Y_{i}^{ (m )} (t )Y_{j}^{ (m )} (t )}. \end{aligned}$$

This completes the description of the system at the first perturbative order.

Now, if we suppose that, for given values of $\sigma_{0}$–$\sigma_{4}$, the perturbative corrections of order higher than one are negligible, Eq. (4.11) can be used to evaluate the probability $\mathscr{P} (t )$ that, at the time instant *t*, all the activation functions in (2.1) can be expanded in a Taylor series according to (3.2). Since $\mathscr{A} (V_{i} )$ can be expanded only if $\vert V_{i}-\mu_{i}\vert < r (\mu_{i} )$, where $r (\mu_{i} )$ is the radius of convergence of the activation function evaluated at the point $V_{i}=\mu_{i}$ (see Appendix A), then $\mathscr{P} (t )$ is defined as follows:

4.13 $$P(t)\overset{\mathrm{def}}{=}\int_{ϒ}p(V,t)\;dV,\;ϒ\overset{\mathrm{def}}{=}\overset{N-1}{\underset{i=0}{⨉}}]\mu_{i}-r(\mu_{i}),\mu_{i}+r(\mu_{i})[$$

where ⨉ represents the Cartesian product of subsets. For a multivariate normal distribution, an analytical expression of $\mathscr {P} (t )$ is not known, therefore it must be evaluated numerically (see Sect. 7). So if $\mathscr{P} (t )\approx1$, we can safely expand the activation function by using Eq. (3.2), therefore under this constraint all the results found in this article are justified. In other terms, this can be considered as a self-consistency check of the theory, which can be further refined if higher-order corrections are taken into account.

## Other Measures of Functional Connectivity

In order to illustrate the generality of our approach, here we briefly describe how it can be extended to compute two other symmetric quantities commonly used to measure the functional connectivity, namely the *mutual information* and the *magnitude-squared coherence* [10, 37, 38].

The mutual information between the membrane potentials (a similar formula holds for the firing rates) of two neurons *i*, *j* is defined as follows:

$$\begin{aligned} \mathcal{I}_{ij} (t ) &\overset{\mathrm{def}}{=}\int _{\mathbb{R}^{2}}p (V_{i},V_{j},t )\log \biggl( \frac {p (V_{i},V_{j},t )}{p (V_{i},t )p (V_{j},t )} \biggr)\,dV_{i}\,dV_{j} \\ &=- \frac{1}{2}\log \bigl(1-\operatorname{Corr}^{2} \bigl(V_{i} (t ),V_{j} (t ) \bigr) \bigr) \end{aligned}$$

where the last identity holds only for normal probability distributions, which is indeed our case. This shows that the mutual information is a simple function that depends trivially on the pairwise correlation between the neurons, which in turn implies that it can be evaluated directly from the results obtained in the previous section.

A similar result holds for the magnitude-squared coherence between the membrane potentials (or the firing rates) of two neurons *i*, *j*. If we call $\mathcal{C}_{ij} (t,\omega )$ the Fourier transform of the time-shifted cross-correlation:

$$\mathcal{C}_{ij} (t,\omega )\overset{\mathrm {def}}{=}\int _{-\infty}^{+\infty}\operatorname{Cov} \bigl(V_{i} (t ),V_{j} (t+s ) \bigr)e^{-\iota\omega s}\,ds $$

(the imaginary unit is denoted by *ι*, to avoid confusion with the neural index *i*), then the magnitude-squared coherence is defined as follows:

$$\operatorname{Coh}_{ij} (t,\omega )\overset{\mathrm {def}}{=} \frac{\mathcal{C}_{ij}^{2} (t,\omega )}{\mathcal {C}_{ii} (t,\omega )\mathcal{C}_{jj} (t,\omega )}. $$

It is straightforward to extend Eqs. (4.4)–(4.6) to include the temporal shift *s*, which allows us to calculate $\operatorname{Cov} (V_{i} (t ),V_{j} (t+s ) )$. This means that the functional connectivity inferred from the correlation, the mutual information and the coherence is qualitatively the same. This further justifies our decision to focus this article only on cross-correlations.

We note that our formalism lends itself in principle also to the calculation of directed asymmetric measures of functional connectivity, such as those based upon transfer entropy [39, 40] or the Granger causality [41–43]. However, an analytical calculation of these directed quantities is beyond the scope of this article.

## Examples

Now we consider some explicit examples of calculation of the correlation structure. First of all, it is important to observe that in this article we consider only cases when the Jacobian matrix $\mathcal{J}$ (see (3.9)) is diagonalizable, so we can calculate the fundamental matrix *Φ* as shown by Eq. (3.11). For this reason we need to know the eigenquantities of $\mathcal{J}$. However, due to the eventual inhomogeneities of the static synaptic weights $\overline{J}_{ij}^{c}$ and of the incoming vertex degree $M_{i}$, and to the non-linearity of the network introduced by the activation function $\mathscr{A}$, in general it is not possible to find a simple relation between the eigenquantities of $\mathcal{J}$ and those of the underlying topology *T*. This means that, even if the matrix *T* has some special structure like circularity or symmetry, in general this cannot be exploited to calculate the eigenquantities of $\mathcal{J}$, because the same structure is not preserved in $\mathcal{J}$ due to the term $\frac{1}{M_{i}}\overline {J}_{ij}^{c}\mathscr{A}' (\mu_{j} )$ (see (3.9) + (3.10)). However, it is important to observe that this is not a problem per se. Actually the method introduced in this article has been applied to the study of relatively generic connectivity matrices, but these results will be published in other papers. For the sake of clarity, here we want to avoid complicated algebraic calculations of the eigenquantities, therefore we will consider the simplest case possible, namely neural networks where the term $\frac{1}{M_{i}}\overline{J}_{ij}^{c}\mathscr {A}' (\mu_{j} )$ does not depend on the indices *i*, *j*. So first of all we suppose that $\overline{J}_{ij}^{c}=\varGamma$$\forall i,j$ and $M_{i}=M$ ∀*i*, where *Γ* is a free parameter that describes the strength of the synaptic connection (if present), while *M* is the number of incoming connections per neuron. Under these assumptions, the condition $\mu_{i}=\mu$ ∀*i* can be satisfied for symmetry reasons if we also suppose that $I_{i}^{c}=I^{c}$ ∀*i*. In this case, from (2.8) + (3.3), it is easy to verify that the parameter *μ* is given by

6.1 $$ \mu=\tau \bigl[\varGamma\mathscr{A} (\mu )+I^{c} \bigr] . $$

Since now the term $\frac{1}{M_{i}}\overline{J}_{ij}^{c}\mathscr {A}' (\mu_{j} )$ does not depend on *i*, *j* anymore, the eigenvalues and eigenvectors of *T*, which we call, respectively, $\lambda_{i}$ and $\mathbf {v}_{i}$, and those of $\mathcal{J}$, respectively, $\widetilde{\lambda}_{i}$ and $\widetilde{\mathbf {v}}_{i}$, are trivially related to each other as follows:

6.2 $$ \begin{aligned} \widetilde{\lambda}_{k}&= - \frac{1}{\tau}+\frac{\varGamma\mathscr {A}' (\mu )}{M}\lambda_{k}, \\ \widetilde{\mathbf {v}}_{k}&= \mathbf {v}_{k}; \end{aligned} $$

therefore now the fundamental matrix *Φ* can be calculated in terms of the eigenquantities of *T*.

It is important to observe that (directed) regular graphs with uniform input satisfy the assumptions above, and for this reason they will be considered from now on, even if the hypothesis of regularity is not strictly required, since we do not need to have also the same number of outgoing connections for each neuron. We also observe that even if under our assumptions the neurons have the same $\overline {J}_{ij}^{c}$, $I_{i}^{c}$ (and, as a consequence, also the same $\mu_{i}$), this does not mean that they all behave identically. For example, from (4.11) we see that the mean of the membrane potentials is $\overline{V}_{i} (t )=\mu+ \sum_{m=3}^{4} \sigma_{m}Y_{i}^{ (m )} (t )$ and that $Y_{i}^{ (3,4 )}$ depend on $\overline{J}^{v} (t )$ and $I^{v} (t )$, which in general are not uniform. This proves that $\overline{V}_{i} (t )$ depends on the index *i*, or in other terms that the neurons are not identical in this network.

To conclude, it is interesting to observe that if we choose $\mathscr {A} (\mu )$ to be the algebraic activation function (see (2.2)), then Eq. (6.1) can be solved analytically, since it can easily be reduced to a fourth-order polynomial equation. Notwithstanding, in every numerical simulation of this article we will use the logistic activation function, since its properties are ideal for studying the phenomenon of stochastic synchronization introduced in Sect. 10. Now we are ready to start with the first example.

### Block-Circulant Matrices with Circulant Blocks

Given two positive integers *F* and *G*, with $1\leq F,G\leq N$, here we suppose that the topology of the network is given by an $N\times N$ block-circulant matrix (with $N=FG$) of the form

$$T=\left [ \textstyle\begin{array}{@{}c@{\quad}c@{\quad}c@{\quad}c@{}} \mathfrak{B}^{ (0 )} & \mathfrak{B}^{ (1 )} & \cdots& \mathfrak{B}^{ (F-1 )} \\ \mathfrak{B}^{ (F-1 )} & \mathfrak{B}^{ (0 )} & \cdots& \mathfrak{B}^{ (F-2 )} \\ \vdots& \vdots& \ddots& \vdots \\ \mathfrak{B}^{ (1 )} & \mathfrak{B}^{ (2 )} & \cdots& \mathfrak{B}^{ (0 )} \end{array}\displaystyle \right ] $$

where $\mathfrak{B}^{ (0 )},\ldots,\mathfrak{B}^{ (F-1 )}$ are $G\times G$ circulant matrices:

$$\mathfrak{B}^{ (i )}=\left [ \textstyle\begin{array}{@{}c@{\quad}c@{\quad}c@{\quad}c@{}} \mathfrak{b}_{0}^{ (i )} & \mathfrak{b}_{1}^{ (i )} & \cdots& \mathfrak{b}_{G-1}^{ (i )} \\ \mathfrak{b}_{G-1}^{ (i )} & \mathfrak{b}_{0}^{ (i )} & \cdots& \mathfrak{b}_{G-2}^{ (i )} \\ \vdots& \vdots& \ddots& \vdots \\ \mathfrak{b}_{1}^{ (i )} & \mathfrak{b}_{2}^{ (i )} & \cdots& \mathfrak{b}_{0}^{ (i )} \end{array}\displaystyle \right ]. $$

All the entries $\mathfrak{b}_{j}^{ (i )}$, for $i=0,\ldots,F-1$ and $j=0,\ldots,G-1$, can only be equal to 0 or 1, with only the exception of $\mathfrak{b}_{0}^{ (0 )}$ that must always be equal to 0 in order to avoid self-connections in the recurrent network. *F* can be interpreted as the number of neural populations, and *G* as the number of neurons per population. Due to this particular structure of the connectivity matrix, all the neurons have the same number of incoming connections *M*, as required.

According to Eqs. (4.4)–(4.6), the correlation structure depends on $\varPhi_{ij} (t )$ and $\sum_{k=0}^{N-1}\varPhi_{ik} (t )\varPhi_{jk} (t )= [\varPhi (t )\varPhi' (t ) ]_{ij}$, therefore now we want to calculate the matrices $\varPhi (t )$ and $\varPhi (t )\varPhi' (t )$ in terms of the eigenquantities of *T*. Since *T* is block-circulant, its eigenvalues are those of the following matrices [44]:

$$\widetilde{\mathfrak{B}}^{ (i )}=\sum_{j=0}^{F-1} \mathfrak{B}^{ (j )}e^{({2\pi}/{F})ij\iota} . $$

In a similar way, since the matrices $\widetilde{\mathfrak{B}}^{ (i )}$ are circulant, we can compute their eigenvalues $\lambda_{j}^{ (i )}$ as follows:

6.3 $$ \lambda_{j}^{ (i )}=\sum _{k=0}^{G-1} \bigl[\widetilde {\mathfrak{B}}^{ (i )} \bigr]_{0k}e^{({2\pi }/{G})jk\iota}=\sum_{k=0}^{G-1} \sum_{l=0}^{F-1}\mathfrak {b}_{k}^{ (l )}e^{2\pi ({jk}/{G}+{il}/{F} )\iota} . $$

Furthermore the matrix of the eigenvectors of *T* is

6.4 $$ \begin{aligned} P= & R_{F}\otimes R_{G}, \\ {} [R_{K} ]_{ij}= & \frac{1}{\sqrt{K}}e^{({2\pi}/{K})ij\iota}, \quad K=F,G, \ i,j=0,\ldots,K-1, \end{aligned} $$

where ⊗ is the Kronecker product of matrices. Since in this article we suppose that the matrix exponential that defines $\varPhi (t )$ could be calculated according to (3.11), we obtain

$$\varPhi (t )\varPhi' (t )=e^{\mathcal{J}t} \bigl( \bigl[e^{\mathcal{J} (t )} \bigr]' \bigr)^{*}=PD (t )P^{*}PD^{*} (t )P^{*}=PD (t )D^{*} (t )P^{*}. $$

∗ is the element-by-element complex conjugation, and $D (t )=\operatorname{diag} (e^{\widetilde{\lambda }_{0}t},\ldots,e^{\widetilde{\lambda}_{N-1}t} )$, where $\widetilde{\lambda}_{k}$ for $k=0,\ldots,N-1$ are the eigenvalues of $\mathcal{J}$ (namely the collection of all the $\widetilde {\lambda}_{j}^{ (i )}$, as given by (6.2) in terms of the $\lambda_{j}^{ (i )}$, for $k=iG+j$). Here we have used the fact that $D (t )$ and *P* are symmetric matrices (see (6.4)) and also the identity:

$$P^{*}P= \bigl(R_{F}^{*}\otimes R_{G}^{*} \bigr) (R_{F}\otimes R_{G} )= \bigl(R_{F}^{*}R_{F} \bigr)\otimes \bigl(R_{G}^{*}R_{G} \bigr)=\mathrm {Id}_{FG}= \mathrm {Id}_{N} $$

due to the mixed-product property of the Kronecker product and to the elementary identity $R_{K}^{*}R_{K}=\mathrm {Id}_{K}$. Now, since

$$\begin{aligned}{} [R_{F}\otimes R_{G} ]_{ij} =& [R_{F} ]_{mn} [R_{G} ]_{pq}= \frac{1}{\sqrt{N}}e^{2\pi ({mn}/{F}+{pq}/{G} )\iota}, \\ m =& \biggl\lfloor \frac{i}{G} \biggr\rfloor , \qquad n= \biggl\lfloor \frac {j}{G} \biggr\rfloor , \qquad p=i-mG, \qquad q=j-nG, \end{aligned}$$

we conclude that

6.5 $$ \begin{aligned} \varPhi_{ij} (t )&= \frac{1}{N} \sum_{k=0}^{N-1} f_{ijk}e^{\widetilde{\lambda}_{k}t}, \\ \bigl[\varPhi (t )\varPhi' (t ) \bigr]_{ij}&= \frac{1}{N} \sum_{k=0}^{N-1} f_{ijk}e^{2\Re (\widetilde {\lambda}_{k} )t} \end{aligned} $$

where $\Re (\widetilde{\lambda}_{k} )=-\frac{1}{\tau }+\frac{\varGamma}{M}\mathscr{A}' (\mu )\Re (\lambda _{k} )$ is the real part of $\widetilde{\lambda}_{k}$, while

$$f_{ijk}\overset{\mathrm{def}}{=}N [R_{F}\otimes R_{G} ]_{ik} [R_{F}\otimes R_{G} ]_{kj}^{*}=e^{2\pi [({1}/{F}) \lfloor {k}/{G} \rfloor ( \lfloor {i}/{G} \rfloor- \lfloor {j}/{G} \rfloor )+({k}/{G}) (i-j ) ]\iota}. $$

We also observe that

6.6 $$ \sum_{j=0}^{N-1} f_{ijk}=N\delta_{0k}; $$

therefore $\sum_{k=0}^{N-1} \varPhi_{ik} (t )=e^{\widetilde {\lambda}_{0}t}$, so from Eqs. (4.4)–(4.6) we obtain

6.7 $$\begin{aligned} &\begin{aligned} \overline{Y_{i}^{ (0 )} (t )Y_{j}^{ (0 )} (t )}={}& \frac{1}{N} \bigl(1-C^{ (0 )} \bigr) \sum _{k=1}^{N-1} f_{ijk}\frac{e^{2\Re (\widetilde {\lambda}_{k} )t}-1}{2\Re (\widetilde{\lambda}_{k} )} \\ &{}+ \biggl[\frac{1}{N}+C^{ (0 )} \biggl(1-\frac{1}{N} \biggr) \biggr]\frac{e^{2\widetilde{\lambda}_{0}t}-1}{2\widetilde{\lambda }_{0}}, \end{aligned} \\ &\begin{aligned} \overline{Y_{i}^{ (1 )} (t )Y_{j}^{ (1 )} (t )}={}& \frac{1}{N} \bigl(1-C^{ (1 )} \bigr) \sum _{k=1}^{N-1} f_{ijk}e^{2\Re (\widetilde{\lambda }_{k} )t} \\ &{}+ \biggl[ \frac{1}{N}+C^{ (1 )} \biggl(1-\frac {1}{N} \biggr) \biggr]e^{2\widetilde{\lambda}_{0}t}, \end{aligned} \\ &\begin{aligned} \overline{Y_{i}^{ (2 )} (t )Y_{j}^{ (2 )} (t )}={}& \mathscr{A}^{2} (\mu ) \Biggl(\frac{1}{MN} \bigl(1-C^{ (2 )} \bigr)\sum_{k=1}^{N-1}f_{ijk} \biggl\vert \frac{e^{\widetilde{\lambda }_{k}t}-1}{\widetilde{\lambda}_{k}}\biggr\vert ^{2} \\ &{}+ \biggl[ \frac {1}{MN}+C^{ (2 )} \biggl(1-\frac{1}{MN} \biggr) \biggr] \biggl(\frac{e^{\widetilde{\lambda}_{0}t}-1}{\widetilde{\lambda }_{0}} \biggr)^{2} \Biggr), \end{aligned} \end{aligned}$$

and finally through (4.10) we obtain an explicit expression for the pairwise correlation structure of the network. It is interesting to observe that Eq. (6.6) is a consequence of the regularity of the graph. Actually, it is well known that $\widetilde{\mathbf {v}}_{0}= (1,\ldots,1 )$ is an eigenvector of any regular graph, and that the other eigenvectors are orthogonal to $\widetilde{\mathbf {v}}_{0}$, so that $\sum_{j=0}^{N-1} [\widetilde{\mathbf {v}}_{k} ]_{j}=0$$\forall k\neq0$. Since *P* is the matrix whose columns are composed of the eigenvectors of $\mathcal{J}$, this means that

6.8 $$ \sum_{j=0}^{N-1}P_{jk}=N \delta_{0k} , $$

of which Eq. (6.6) is a particular case.

**Fig. 2:**
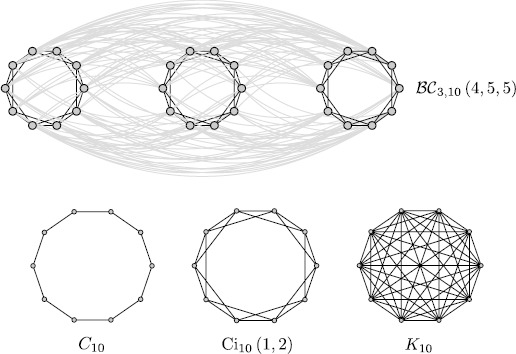
Examples of the block-circulant graphs for different values of *F* and *ξ*, with *G* fixed. *The figure on the top* represents the case $\mathcal {BC}_{3,10} (4,5,5 )$, obtained for $F=3$, $G=10$, and $\xi_{0}=\xi_{1}=\xi_{2}=2$. *The figure at the bottom* shows some examples of the special case $\operatorname{Ci}_{N} (1,2,\ldots,\xi )=\mathcal {BC}_{1,N} (2\xi -H (\xi- \lfloor\frac{N}{2} \rfloor+ (-1 )^{N} ) )$ (*circulant graph*) for $N=10$, namely $C_{N}=\operatorname {Ci}_{N} (1 )$ (*cyclic graph*), $\operatorname{Ci}_{N} (1,2 )$, and finally $K_{N}=\operatorname{Ci}_{N} (1,2,\ldots, \lfloor\frac {N}{2} \rfloor )$ (*complete graph*)

Now we show an explicit example of this technique, namely the case when the blocks of the matrix *T* have the following symmetric circulant band structure:

6.9 $$\begin{aligned} &\mathfrak{B}^{ (i )}=\left [ \textstyle\begin{array}{@{}c@{\quad}c@{\quad}c@{\quad}c@{\quad}c@{\quad }c@{\quad}c@{\quad}c@{\quad}c@{\quad}c@{}} 1-\delta_{i0} & 1 & \cdots& 1 & 0 & \cdots& 0 & 1 & \cdots& 1 \\ 1 & 1-\delta_{i0} & \ddots& & \ddots& \ddots& & \ddots& \ddots& \vdots \\ \vdots& \ddots& \ddots& \ddots& & \ddots& \ddots& & \ddots& 1 \\ 1 & & \ddots& \ddots& \ddots& & \ddots& \ddots& & 0 \\ 0 & \ddots& & \ddots& \ddots& \ddots& & \ddots& \ddots& \vdots \\ \vdots& & \ddots& & \ddots& \ddots& \ddots& & \ddots& 0 \\ 0 & & & \ddots& & \ddots& \ddots& \ddots& & 1 \\ 1 & \ddots& & & \ddots& & \ddots& \ddots& \ddots& \vdots \\ \vdots& \ddots& \ddots& & & \ddots& & \ddots& 1-\delta_{i0} & 1 \\ 1 & \cdots& 1 & 0 & \cdots& 0 & 1 & \cdots& 1 & 1-\delta_{i0} \end{array}\displaystyle \right ] \end{aligned}$$

where, supposing for simplicity that $G\geq3$, the first row of $\mathfrak{B}^{ (i )}$ (excluding the term $\mathfrak{b}_{0}^{ (i )}$, which is 0 for $i=0$ and 1 for $i>0$) can be written explicitly as

$$\begin{aligned} \textstyle\begin{cases} \mathfrak{b}_{j}^{ (i )}= 1, & (1\leq j\leq\xi_{i} )\vee (\varrho_{i}\leq j\leq G-1 ), \\ \mathfrak{b}_{j}^{ (i )}= 0, & \xi_{i}< j< \varrho_{i}, \end{cases}\displaystyle \displaystyle \displaystyle \\ \begin{aligned} \varrho_{i}&= G-\xi_{i}+H \biggl(\xi_{i}- \biggl\lfloor \frac {G}{2} \biggr\rfloor + (-1 )^{G} \biggr), \\ H (x )&= \textstyle\begin{cases} 0, & x\leq0, \\ 1, & x>0, \end{cases}\displaystyle \displaystyle \displaystyle \end{aligned} \end{aligned}$$

where $1\leq\xi_{i}\leq \lfloor\frac{G}{2} \rfloor$, while $H (\cdot )$ is the Heaviside step function. Here we have to suppose that $G\geq3$ because otherwise it is not possible to distinguish the diagonal band from the corner elements. Now, the bandwidth of $\mathfrak{B}^{ (i )}$ is $2\xi_{i}+1$, so this defines the integer parameters $\xi_{i}$. Moreover, $\mathcal {M}_{0}\overset{\mathrm{def}}{=}2\xi_{0}-H (\xi_{0}- \lfloor\frac{G}{2} \rfloor+ (-1 )^{G} )$ represents the number of connections that every neuron in a given population receives from the neurons in the same population. Instead $\mathcal{M}_{i}\overset{\mathrm{def}}{=}2\xi_{i}+1-H (\xi _{i}- \lfloor\frac{G}{2} \rfloor+ (-1 )^{G} )$, for $i=1,\ldots,F-1$, is the number of connections that every neuron in the *k*th population receives from the neurons in the $(i+k )$th mod*F* population, for $k=0,\ldots,F-1$. So the total number of incoming connections per neuron is $M=F-1+\sum_{i=0}^{F-1} [2\xi_{i}-H (\xi_{i}- \lfloor\frac{G}{2} \rfloor + (-1 )^{G} ) ]$. The graph with this special block-circulant adjacency matrix will be represented by the notation $\mathcal{BC}_{F,G} (\mathcal {M}_{0},\ldots,\mathcal{M}_{F-1} )$, and some examples are shown in Fig. 2 for different values of *F* and *ξ*. This can be considered as a toy model for describing a network of *F* cortical columns containing *G* neurons each. The parameters $\xi_{i}$ can be adjusted in order to generate $\mathcal{M}_{0}$ local and $\mathcal{M}_{i}$ long-range connections compatible with recent neuroanatomical studies [45], providing a rough description of a wide area of neural tissue. This idea will be extended to the case of irregular graphs in Sect. 6.3.2. Moreover, it is important to observe that even if in this case all the matrices $\mathfrak{B}^{ (i )}$ are symmetric, the matrix *T* is not, since the number of connections in every block is different (the case of symmetric connectivity matrices is studied in Sect. 6.2).

Now, by using Eqs. (6.3) + (6.9), we obtain

6.10 $$ \begin{aligned} \lambda_{mG+n}&= \textstyle\begin{cases} F-1+ \sum_{k=0}^{F-1} g (n,\xi_{k},G ), & m=0, \forall n, \\ -1+ \sum_{k=0}^{F-1} e^{({2\pi}/{F})mk\iota}g (n,\xi _{k},G ), & m\neq0, \forall n, \end{cases}\displaystyle \\ g (n,\xi_{k},G )&= \textstyle\begin{cases} 2\xi_{k}-H (\xi_{k}- \lfloor\frac{G}{2} \rfloor + (-1 )^{G} ), & n=0, \forall\xi_{k}, \\ -1, & n\neq0, \xi_{k}= \lfloor\frac{G}{2} \rfloor, \\ \frac{\sin ({\pi n (2\xi_{k}+1 )}/{G} )}{\sin ({\pi n}/{G} )}-1, & n\neq0, \xi_{k}< \lfloor\frac{G}{2} \rfloor, \end{cases}\displaystyle \displaystyle \displaystyle \end{aligned} $$

with $m=0,\ldots,F-1$ and $n=0,\ldots,G-1$. In general a closed form for $\sum_{k=0}^{F-1} g (n,\xi_{k},G )$ and $\sum_{k=0}^{F-1} e^{({2\pi}/{F})mk\iota}g (n,\xi _{k},G )$ is not known, since it depends on the sequence $(\xi_{0},\ldots ,\xi_{F-1} )$.

However, many different special cases can be studied. The simplest one is obtained for $\xi_{0}=\cdots=\xi_{F-1}\overset {\mathrm{def}}{=}\xi$ (see the example $\mathcal{BC}_{3,10} (4,5,5 )$ in Fig. 2, obtained for $F=3$, $G=10$, and $\xi_{0}=\xi_{1}=\xi_{2}=2$), and in this case Eq. (6.10) gives:

6.11 $$ \lambda_{mG+n}= \textstyle\begin{cases} F-1+Fg (n,\xi,G ), & m=0, \forall n, \\ -1, & m\neq0, \forall n, \end{cases} $$

with $M=F-1+F [2\xi-H (\xi- \lfloor\frac{G}{2} \rfloor+ (-1 )^{G} ) ]$. Therefore in this case all the eigenvalues are real, as it must be, since with this special choice of the parameters the matrix *T* is symmetric. For $F=1$ and $\xi< \lfloor\frac{N}{2} \rfloor$ we have $M=2\xi$ and Eq. (6.11) gives the eigenvalues of the circulant graph (see the example $\operatorname{Ci}_{N} (1,2,\ldots,\xi )$ in Fig. 2):

6.12 $$ \lambda_{n}= \textstyle\begin{cases} 2\xi, & n=0, \\ \frac{\sin ({\pi n (2\xi+1 )}/{N} )}{\sin ({\pi n}/{N} )}-1, & n\neq0. \end{cases} $$

Now, the cyclic graph $C_{N}$ is obtained in the special case $\xi=1$, and due to the Dirichlet kernel identity, (6.12) reduces to:

6.13 $$ \lambda_{n}=2\cos \biggl(\frac{2\pi n}{N} \biggr) . $$

Instead for $\xi= \lfloor\frac{G}{2} \rfloor$ (full band) and $\forall F,G$ we have $M=N-1$ and Eq. (6.11) gives the eigenvalues of the complete graph $K_{N}$:

6.14 $$ \lambda_{n}= \textstyle\begin{cases} N-1, & n=0, \\ -1, & n\neq0. \end{cases} $$

By replacing Eqs. (6.10)–(6.14) in (6.7), we obtain the pairwise correlation structure of the corresponding network topology. In general there is no closed form for the finite sums in (6.7), with only the exception of the complete graph, for which we obtain

6.15 $$\begin{aligned} &\begin{aligned} \overline{Y_{i}^{ (0 )} (t )Y_{j}^{ (0 )} (t )}={}& \biggl[\frac{1}{N}+C^{ (0 )} \biggl(1-\frac{1}{N} \biggr) \biggr]\frac{e^{2\widetilde{\lambda }_{0}t}-1}{2\widetilde{\lambda}_{0}} \\ &{}+ \bigl(1-C^{ (0 )} \bigr) \biggl( \delta_{ij}-\frac{1}{N} \biggr)\frac{e^{2\widetilde {\lambda}_{1}t}-1}{2\widetilde{\lambda}_{1}}, \end{aligned} \\ &\begin{aligned} \overline{Y_{i}^{ (1 )} (t )Y_{j}^{ (1 )} (t )}={}& \biggl[\frac{1}{N}+C^{ (1 )} \biggl(1-\frac{1}{N} \biggr) \biggr]e^{2\widetilde{\lambda }_{0}t} \\ &{}+ \bigl(1-C^{ (1 )} \bigr) \biggl( \delta_{ij}-\frac {1}{N} \biggr)e^{2\widetilde{\lambda}_{1}t}, \end{aligned} \\ &\begin{aligned} \overline{Y_{i}^{ (2 )} (t )Y_{j}^{ (2 )} (t )}={}& \frac{\mathscr{A}^{2} (\mu )}{N-1} \biggl\{ \biggl[\frac{1}{N}+C^{ (2 )} \biggl(N-1-\frac{1}{N} \biggr) \biggr] \biggl(\frac{e^{\widetilde{\lambda }_{0}t}-1}{\widetilde{\lambda}_{0}} \biggr)^{2} \\ &{}+ \bigl(1-C^{ (2 )} \bigr) \biggl( \delta_{ij}-\frac{1}{N} \biggr) \biggl(\frac {e^{\widetilde{\lambda}_{1}t}-1}{\widetilde{\lambda}_{1}} \biggr)^{2} \biggr\} \end{aligned} \end{aligned}$$

where

$$\widetilde{\lambda}_{0}=-\frac{1}{\tau}+\varGamma \mathscr{A}' (\mu ), \qquad\widetilde{\lambda}_{1}=- \frac{1}{\tau}-\frac {\varGamma}{N-1}\mathscr{A}' (\mu ). $$

Some interesting consequences of these formulas, for the complete graph and other kinds of topologies, will be analyzed in Sects. 8, 9, 10. However, before that, in the next section we want to show the effectiveness of this perturbative method by applying it to another class of topologies, that of symmetric connectivity matrices.

### Symmetric Matrices

Another case where the matrices $\varPhi (t )$ and $\varPhi (t )\varPhi' (t )$ can be computed easily is when *T* is a general symmetric matrix. Since its entries are real, it can be diagonalized by an orthogonal matrix *P* (namely a matrix such that $P^{-1}=P'$), therefore we have $\varPhi (t )=PD (t )P'$. Since in this case the matrix $\mathcal{J}$ is symmetric, we also obtain

$$\varPhi (t )\varPhi' (t )=e^{2\mathcal {J}t}=PD^{2} (t )P' $$

and so

6.16 $$ \begin{aligned} \varPhi_{ij} (t )&= \sum _{k=0}^{N-1} P_{ik}P_{jk}e^{\widetilde{\lambda}_{k}t}, \\ \bigl[\varPhi (t )\varPhi' (t ) \bigr]_{ij}&= \sum _{k=0}^{N-1} P_{ik}P_{jk}e^{2\widetilde{\lambda}_{k}t}. \end{aligned} $$

**Fig. 3:**
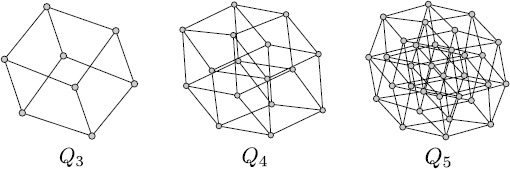
Three examples of the hypercube $Q_{n}$

Now we show an explicit example, by applying equations in (6.16) to the case when the neurons are connected according to a hypercube graph $Q_{n}$. The hypercube can be defined in terms of the Cartesian product of graphs [46] (see also Sect. 6.3.1):
6.17 where *n* is an integer and $K_{2}$ is the complete graph with 2 vertices. Some examples of $Q_{n}$ for different values of *n* are shown in Fig. 3. Clearly in this case $M=n$, and from (6.17) and by definition of the Cartesian product, the topology of the hypercube can be expressed as follows:

6.18 $$ \begin{aligned} T_{Q_{1}} & =\left [ \textstyle\begin{array}{@{}c@{\quad}c@{}} 0 & 1\\ 1 & 0 \end{array}\displaystyle \right ], \\ T_{Q_{n}} & = \left [ \textstyle\begin{array}{@{}c@{\quad}c@{}} T_{Q_{n-1}} & \mathrm {Id}_{2^{n-1}} \\ \mathrm {Id}_{2^{n-1}} & T_{Q_{n-1}} \end{array}\displaystyle \right ], \quad n\geq2. \end{aligned} $$

It is easy to check that the eigenvalues of the matrix $T_{Q_{n}}$ are $n-2m$, for $m=0,\ldots,n$ and with algebraic multiplicity $\bigl({\scriptsize\begin{matrix}n\cr m\end{matrix}} \bigr) $. If we rewrite these eigenvalues with the following order:

$$\lambda_{k}= \sum_{l=0}^{n-1} (-1 )^{ \lfloor {k}/{2^{l}} \rfloor}, \quad k=0,\ldots,N-1, $$

then the corresponding eigenvectors are the columns of the matrix:

6.19 $$ P=\frac{1}{\sqrt{N}}H_{N} $$

where $H_{N}$ is an $N\times N$ Hadamard matrix, defined as follows:

6.20 $$\begin{aligned} H_{1} &= [1 ], \\ H_{2} &=\left [ \textstyle\begin{array}{@{}c@{\quad}c@{}} 1& 1 \\ 1 &-1 \end{array}\displaystyle \right ], \\ H_{2^{n}} &= H_{2}\otimes H_{2^{n-1}}, \quad n\geq2. \end{aligned}$$

From the property $H_{N}H_{N}'=N\mathrm {Id}_{N}$ it is clear that the matrix *P* defined by (6.19) is orthogonal. Now, from (6.19) + (6.20) we obtain

$$\begin{aligned} P_{ik}P_{jk} =&\frac{1}{N}\mathfrak{f}_{ijk}, \\ \mathfrak{f}_{ijk} \overset{\mathrm{def}}{=}& (-1 )^{\mathscr{S}_{ijk}}, \\ \mathscr{S}_{ijk} \overset{\mathrm{def}}{=}& \sum _{l=0}^{n-1} \bigl(h_{l} (i )-h_{l} (j ) \bigr)h_{l} (k ), \\ h_{l} (x ) \overset{\mathrm{def}}{=}& \biggl\lfloor \frac {x}{2^{n-l-1}} \biggr\rfloor -2 \biggl\lfloor \frac{x}{2^{n-l}} \biggr\rfloor . \end{aligned}$$

The reader can check that $\sum_{j=0}^{N-1} (-1 )^{\mathscr{S}_{ijk}}=N\delta_{0k}$, as it must be according to (6.8), so we get

6.21 $$\begin{aligned} &\begin{aligned} \overline{Y_{i}^{ (0 )} (t )Y_{j}^{ (0 )} (t )}={}& \frac{1}{N} \bigl(1-C^{ (0 )} \bigr) \sum _{k=1}^{N-1} \mathfrak{f}_{ijk} \frac{e^{2\widetilde {\lambda}_{k}t}-1}{2\widetilde{\lambda}_{k}} \\ &{}+ \biggl[\frac {1}{N}+C^{ (0 )} \biggl(1- \frac{1}{N} \biggr) \biggr]\frac {e^{2\widetilde{\lambda}_{0}t}-1}{2\widetilde {\lambda}_{0}}, \end{aligned} \\ &\begin{aligned} \overline{Y_{i}^{ (1 )} (t )Y_{j}^{ (1 )} (t )}={}&\frac{1}{N} \bigl(1-C^{ (1 )} \bigr) \sum _{k=1}^{N-1} \mathfrak{f}_{ijk}e^{2\widetilde {\lambda}_{k}t} \\ &{}+ \biggl[\frac{1}{N}+C^{ (1 )} \biggl(1-\frac {1}{N} \biggr) \biggr]e^{2\widetilde{\lambda}_{0}t}, \end{aligned} \\ &\begin{aligned} \overline{Y_{i}^{ (2 )} (t )Y_{j}^{ (2 )} (t )}={}& \mathscr{A}^{2} (\mu ) \Biggl(\frac{1}{nN} \bigl(1-C^{ (2 )} \bigr)\sum_{k=1}^{N-1} \mathfrak{f}_{ijk} \biggl(\frac{e^{\widetilde{\lambda }_{k}t}-1}{\widetilde{\lambda}_{k}} \biggr)^{2} \\ &{}+ \biggl[\frac {1}{nN}+C^{ (2 )} \biggl(1-\frac{1}{nN} \biggr) \biggr] \biggl(\frac{e^{\widetilde{\lambda}_{0}t}-1}{\widetilde{\lambda }_{0}} \biggr)^{2} \Biggr). \end{aligned} \end{aligned}$$

We observe that Eqs. (6.7) and (6.21) are very similar. This is clearly a consequence of the regularity of the corresponding graphs.

### Examples with More Complex Connectivity Matrices

Now we briefly discuss some more complex examples of connectivity. In particular, in Sect. 6.3.1 we focus on examples of more complex regular graphs, while in Sect. 6.3.2 we relax the hypothesis of regularity.

#### Product of Regular Graphs

In Sects. 6.1 and 6.2 we showed some relatively simple examples of regular graphs. It is possible to build more complicated topologies by means of graph operations that transform a graph into another while allowing us to calculate easily the spectrum of the new graph from that of the old one. There are two main kinds of graph operations: unary and binary. An example of unary operation is the graph complement that transforms a graph $\mathcal{G}$ into its complement $\overline{\mathcal{G}}$, namely in the graph with the same vertices of $\mathcal{G}$ and such that two distinct vertices of $\overline{\mathcal{G}}$ are connected if and only if they are disconnected in $\mathcal{G}$. For example, the complement of $C_{4}$ is the disjoint union of two graphs $K_{2}$. On the other side, binary operations create a new graph from two initial graphs $\mathcal {G}$, $\mathcal{H}$. In this section we discuss only graph products, namely a particular kind of binary operations that prove very useful for studying networks made of different interconnected populations.

In all the examples that follow, the new graph resulting from the product of $\mathcal{G}$ and $\mathcal{H}$ has a vertex set $\mathcal {V} (\mathcal{G} )\times\mathcal{V} (\mathcal {H} )$, where × is the Cartesian product of sets and $\mathcal{V} (\mathcal{G} )$, $\mathcal{V} (\mathcal{H} )$ represent the collection of vertices of $\mathcal{G}$, $\mathcal{H}$, respectively. A well-known example that has already been introduced in Sect. 6.2 is the Cartesian product $\mathcal{G}\,\square \,\mathcal{H}$. This represents a new graph, where any two vertices $(g,h )$ and $(g',h' )$ in $\mathcal{V} (\mathcal{G} )\times\mathcal{V} (\mathcal{H} )$ are connected if and only if either $g=g'$ and *h* is connected to $h'$ in $\mathcal{H}$, or $h=h'$ and *g* is connected to $g'$ in $\mathcal{G}$. Due to this rule, $\mathcal{G}\,\square \,\mathcal{H}$ has the following topology:

$$T_{\mathcal{G}\square \mathcal{H}}=T_{\mathcal{G}}\otimes \mathrm {Id}_{N_{\mathcal{H}}}+ \mathrm {Id}_{N_{\mathcal {G}}}\otimes T_{\mathcal{H}} $$

where, as usual, ⊗ is the Kronecker product of matrices, and $N_{\mathcal{G}}$, $N_{\mathcal{H}}$ are the number of vertices of $\mathcal{G}$, $\mathcal{H}$, respectively. From this definition and by means of the mixed-product property of the Kronecker product, it is easy to prove that, if we call $\lambda_{i}^{\mathcal{G}}$ (for $i=0,\ldots,N_{\mathcal{G}}-1$), $\lambda_{j}^{\mathcal{H}}$ (for $j=0,\ldots,N_{\mathcal{H}}-1$) the eigenvalues of $\mathcal {G}$, $\mathcal{H}$, respectively, then the eigenvalues of $\mathcal{G}\,\square \,\mathcal{H}$ are $\lambda_{i}^{\mathcal{G}}+\lambda_{j}^{\mathcal{H}}$ for all the possible pairs $(i,j )$. Moreover, if $\mathbf {v}_{i}^{\mathcal{G}}$, $\mathbf {v}_{j}^{\mathcal{H}}$ are the corresponding eigenvectors, it is straightforward to prove that the eigenvectors of $\mathcal{G}\,\square \,\mathcal{H}$ are $\mathbf {v}_{i}^{\mathcal{G}}\otimes \mathbf {v}_{j}^{\mathcal{H}}$ for all $(i,j )$. This result is true for every pair of graphs that are combined through the Cartesian product. However, if $\mathcal{G}$, $\mathcal{H}$ are regular with vertex degrees $M_{\mathcal{G}}$, $M_{\mathcal{H}}$, respectively, then $\mathcal{G}\,\square \,\mathcal{H}$ is also regular, with degree $M_{\mathcal{G}}+M_{\mathcal{H}}$. This is a consequence of the fact that a graph is regular if and only if $(1,\ldots ,1 )$ is an eigenvector (with the vertex degree as corresponding eigenvalue), and the fact that the tensor product $\mathbf {v}_{i}^{\mathcal {G}}\otimes \mathbf {v}_{j}^{\mathcal{H}}$ between two all-ones vectors $\mathbf {v}_{i}^{\mathcal {G}}$, $\mathbf {v}_{j}^{\mathcal{H}}$ is itself an all-ones vector with $\lambda_{i}^{\mathcal{G}}+\lambda _{j}^{\mathcal{H}} = M_{\mathcal{G}}+M_{\mathcal{H}}$ as corresponding eigenvalue. Therefore we conclude that, given graphs with known spectra, it is possible to build more complex graphs whose spectra are easily determined through the rules shown above. This proves that the theory introduced in this article can easily be used to calculate analytically the correlation structure of neural networks with highly complex connectivity matrices. Typical examples of the Cartesian product are the hypercube (see Eq. (6.17)), the circular ladder $\operatorname{CL} _{N}=C_{N}\,\square \, K_{2}$ (also known as *prism graph*), the *d*-dimensional torus $\mathscr {T} (N_{0},\ldots,N_{d-1} )=C_{N_{0}}\,\square \cdots \square \, C_{N_{d-1}}$, and so on.

Another case is the tensor product $\mathcal{G}\otimes\mathcal{H}$, where any two vertices $(g,h )$ and $(g',h' )$ are connected if and only if *g* is connected to $g'$ in $\mathcal{G}$ and *h* is connected to $h'$ in $\mathcal{H}$. From this rule we get the following topology:

$$T_{\mathcal{G}\otimes\mathcal{H}}=T_{\mathcal{G}}\otimes T_{\mathcal{H}} $$

so it follows that the eigenvalues of $\mathcal{G}\otimes \mathcal{H}$ are $\lambda_{i}^{\mathcal{G}}\lambda_{j}^{\mathcal{H}}$ for all $(i,j )$, while $\mathbf {v}_{i}^{\mathcal{G}}\otimes \mathbf {v}_{j}^{\mathcal{H}}$ are their corresponding eigenvectors. Again, this result is true for any $\mathcal{G}$, $\mathcal{H}$, but if the graphs are both regular, then $\mathcal{G}\otimes\mathcal{H}$ is also regular, with vertex degree $M_{\mathcal{G}}M_{\mathcal{H}}$. An example of tensor product is the crown graph $S_{N}^{0}=K_{N}\otimes K_{2}$.

Now we consider the strong product $\mathcal{G}\boxtimes\mathcal{H}$, where any two vertices $(g,h )$ and $(g',h' )$ are connected whenever *g* and $g'$ are equal or connected in $\mathcal{G}$, and *h* and $h'$ are equal or connected in $\mathcal{H}$. So we get

$$T_{\mathcal{G}\boxtimes\mathcal{H}}= (T_{\mathcal {G}}+\mathrm {Id}_{N_{\mathcal{G}}} )\otimes (T_{\mathcal {H}}+\mathrm {Id}_{N_{\mathcal{H}}} )-\mathrm {Id}_{N_{\mathcal{G}}N_{\mathcal{H}}} . $$

From this formula it follows that the eigenvalues of $\mathcal{G}\boxtimes\mathcal{H}$ are $(\lambda_{i}^{\mathcal{G}}+1 ) (\lambda _{j}^{\mathcal{H}}+1 )-1$ for all $(i,j )$, while $\mathbf {v}_{i}^{\mathcal {G}}\otimes \mathbf {v}_{j}^{\mathcal{H}}$ are their corresponding eigenvectors. Again, this result is true for any $\mathcal{G}$, $\mathcal{H}$, but if the graphs are both regular, then $\mathcal{G}\boxtimes\mathcal{H}$ is also regular, with vertex degree $(M_{\mathcal{G}}+1 ) (M_{\mathcal {H}}+1 )-1$. A trivial example is $K_{N_{\mathcal{G}}+N_{\mathcal {H}}}=K_{N_{\mathcal{G}}}\boxtimes K_{N_{\mathcal{H}}}$, from which it is possible to prove in an alternative way Eq. (6.14) by iteration.

Finally, we show the lexicographic product $\mathcal{G}\bullet \mathcal{H}$, where any two vertices $(g,h )$ and $(g',h' )$ are connected if and only if either *g* is connected to $g'$ in $\mathcal{G}$, or $g=g'$ and *h* is connected to $h'$ in $\mathcal{H}$. Therefore the topology matrix is

$$T_{\mathcal{G}\bullet\mathcal{H}}=T_{\mathcal{G}}\otimes\mathbb {I}_{N_{\mathcal{H}}}+ \mathrm {Id}_{N_{\mathcal{G}}}\otimes T_{\mathcal{H}} $$

where $\mathbb{I}_{N_{\mathcal{H}}}$ is the $N_{\mathcal {H}}\times N_{\mathcal{H}}$ all-ones matrix. In general there is no simple expression for the spectrum of $\mathcal{G}\bullet\mathcal{H}$. However, if $\mathcal{H}$ is regular, from (6.8) it is easy to prove that $\lambda_{i}^{\mathcal{G}}N_{\mathcal {H}}+M_{\mathcal{H}}$ and $\lambda_{j}^{\mathcal{H}}$ are eigenvalues of $\mathcal {G}\bullet\mathcal{H}$, with eigenvectors $\mathbf {v}_{i}^{\mathcal{G}}\otimes \mathbf {v}_{0}^{\mathcal{H}}$ and $\mathbf {v}_{i}^{\mathcal{G}}\otimes \mathbf {v}_{j}^{\mathcal{H}}$ (for $j>0$), respectively, where $\mathbf {v}_{0}^{\mathcal {H}}= (1,\ldots,1 )$. If also $\mathcal{G}$ is regular, then $\mathcal{G}\bullet\mathcal{H}$ is regular with vertex degree $M_{\mathcal{G}}N_{\mathcal {H}}+M_{\mathcal{H}}$. An example of lexicographic product is the so called *double graph* of $\mathcal{G}$, namely $\mathcal{D} [\mathcal{G} ]=\mathcal{G}\bullet\overline{K}_{2}$ [47], where $\overline{K}_{2}$ is the complement of $K_{2}$, or in other terms the graph on 2 vertices without edges.

**Fig. 4:**
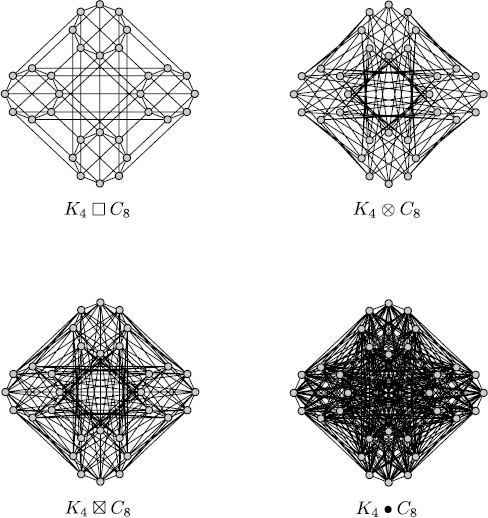
Examples of graph products: $K_{4}\,\square \, C_{8}$ (*top-left panel*), $K_{4}\otimes C_{8}$ (*top-right*), $K_{4}\boxtimes C_{8}$ (*bottom-left*), $K_{4}\bullet C_{8}$ (*bottom-right*). The figure shows the differences between the different products, in particular the number of connections per neuron, which is, respectively, $M_{K_{4}}+M_{C_{8}}=5$, $M_{K_{4}}M_{C_{8}}=6$, $(M_{K_{4}}+1 ) (M_{C_{8}}+1 )-1=11$, $M_{K_{4}}N_{C_{8}}+M_{C_{8}}=26$ (some connections may be overlapping). In general, a product between two graphs $\mathcal{G}$, $\mathcal{H}$ can be interpreted as a system of $N_{\mathcal{G}}$ neural populations with $N_{\mathcal{H}}$ neurons each, interconnected in different ways according to the graph product that has been chosen

A more complex example of the graph products introduced so far is shown in Fig. 4. This example clearly shows that the products can be used to generate easily networks with sub-populations connected in different ways, increasing the biological plausibility of the connectivity matrix. In other terms, this can be interpreted as a way to build more complex connections between the neural populations compared to the case $\mathcal{BC}_{F,G} (\mathcal{M}_{0},\ldots,\mathcal{M}_{F-1} )$ studied in Sect. 6.1. We conclude by observing that it is also possible to define many other kinds of products, which are not considered here. The interested reader is referred to the literature.

#### Irregular Graphs

Up to now we have studied only regular graphs, because for this class it is possible to calculate easily the eigenquantities of $\mathcal{J}$ from those of *T* by means of Eq. (6.2). In this section we show that this is not a strict requirement of our theory and that it can be applied also to irregular graphs. Regularity can be broken in two different ways: either by introducing non-uniform weights (since, by definition, regular graphs are unweighted), or by considering vertices with different (incoming or outgoing) degrees. In both cases we show how to calculate the eigenquantities of the Jacobian matrix in a relatively simple way.

First of all, in Sect. 6 we observed that Eq. (6.2) could be applied more widely also to irregular graphs with uniform weights and the same number of incoming connections, but with different outgoing connections for each neuron. In this section we generalize that result. Indeed, if for a given collection of input currents, we consider those graphs with a generally irregular topology *T* and a non-uniform weight matrix $\overline{J}^{c}$ such that the ratio $\mathcal{R}\overset{\mathrm{def}}{=}\frac {\mathscr{A}' (\mu_{j} )}{M_{i}}$ does not depend on the indices *i*, *j*, we can easily see that $\mathcal {J}_{ij}=\mathcal{R}T_{ij}\overline{J}_{ij}^{c}$. Therefore for this class of graphs the eigenquantities of the Jacobian matrix depend trivially on those of the (unperturbed) weight matrix $T\circ\overline{J}^{c}$ (here ∘ represents the Hadamard product of matrices), which are supposed to be known. An example of such connectivity is represented by the *ring model* of Hansel and Sompolinsky [48], which is a well-known model for feature selectivity in primary visual cortex. In this model each cortical hypercolumn is modeled as a collection of *F* minicolumns with *G* neurons each that respond to a particular feature of the stimulus, namely the orientation of bars in the visual scene. If we introduce the function $\mathfrak{p} (\cdot )$, which maps each neuron *i* to the minicolumn it belongs to, then we call $\theta_{\mathfrak {p} (i )}$ the preferred orientation of that neuron. In this way all the neurons in the same minicolumn have the same preferred orientation. According to experimental evidence, Hansel and Sompolinsky proposed the following connectivity matrix for the hypercolumn, where the strength of the synaptic interaction between two neurons depends on the difference between their preferred orientations:

$$T_{ij}\overline{J}_{ij}^{c}=\varGamma+\varDelta \cos \bigl(2 (\theta _{\mathfrak{p} (i )}-\theta_{\mathfrak{p} (j )} ) \bigr). $$

Here *Γ*, *Δ* are free parameters that define the strength of the synaptic connections within and among the minicolumns. We also observe that this formula defines a non-uniform weight matrix, therefore the corresponding graph is irregular. Now, in the primary visual cortex the preferred orientations are organized in a circular scheme around special points of the orientation map, known as *pinwheels*, in order to represent all the possible bar orientations in the range $[0,\pi )$. For this reason, we can choose $\theta _{\mathfrak{p} (i )}=\vartheta+\frac{\pi}{F} \lfloor\frac{i}{G} \rfloor$ where *ϑ* is an arbitrary angle, so the connectivity matrix of the system can be rewritten as follows:

$$T\circ\overline{J}^{c}= \left[ \textstyle\begin{array}{@{}c@{\quad}c@{\quad}c@{\quad}c@{}} \overline{\mathfrak{B}}^{ (0 )} & \overline{\mathfrak {B}}^{ (1 )} & \cdots& \overline{\mathfrak{B}}^{ (F-1 )} \\ \overline{\mathfrak{B}}^{ (F-1 )} & \overline{\mathfrak {B}}^{ (0 )} & \cdots& \overline{\mathfrak{B}}^{ (F-2 )} \\ \vdots& \vdots& \ddots& \vdots \\ \overline{\mathfrak{B}}^{ (1 )} & \overline{\mathfrak {B}}^{ (2 )} & \cdots& \overline{\mathfrak{B}}^{ (0 )} \end{array}\displaystyle \right] $$

where $\overline{\mathfrak{B}}^{ (0 )},\ldots,\overline {\mathfrak{B}}^{ (F-1 )}$ are $G\times G$ matrices (with $FG=N$) of the form

$$\overline{\mathfrak{B}}^{ (k )}=\mathfrak{J}_{k} \left[ \textstyle\begin{array}{@{}c@{\quad}c@{\quad}c@{\quad}c@{}} 1-\delta_{0k} & 1 & \cdots& 1 \\ 1 & 1-\delta_{0k} & \cdots& 1 \\ \vdots& \vdots& \ddots& \vdots \\ 1 & 1 & \cdots& 1-\delta_{0k} \end{array}\displaystyle \right],\quad\mathfrak{J}_{k}=\varGamma+ \varDelta \cos \biggl(\frac{2\pi }{F}k \biggr), $$

for $k=0,\ldots,F-1$. In [48] the authors also considered an external current of the form $I_{i}=\mathcal{C} [1-\varepsilon+\varepsilon\cos (2 (\theta_{\mathfrak{p} (i )}-\widetilde{\theta} ) ) ]$, where $\mathcal{C}$ is the maximum amplitude of the external input, $0\leq\varepsilon\leq0.5$ measures the degree of modulation of $I_{i}$, and $\widetilde{\theta}$ is the orientation for which the external input is maximum. We also observe that, if *ε* is small enough, $\varepsilon [-1+\cos (2 (\theta_{\mathfrak {p} (i )}-\widetilde{\theta} ) ) ]$ in the formula of $I_{i}$ can be interpreted as the term $\sigma_{4}I_{i}^{v}$ in Eq. (2.9), so that we can identify $I_{i}^{c}=\mathcal{C}$ ∀*i*.

Clearly this is an extension to the irregular case of the topology studied in Sect. 6.1, which can be re-obtained for $\varDelta =0$. It is easy to verify that $M_{i}=N-1$ ∀*i* (so in this case the topology *T* is regular, but the graph is not, due to the non-uniform weight matrix $\overline{J}^{c}$), and, moreover,

$$\mathcal{S}\overset{\mathrm{def}}{=}\sum_{j=0}^{N-1}T_{ij} \overline {J}_{ij}^{c}= (G-1 )\mathfrak{J}_{0}+G \sum_{j=1}^{F-1}\mathfrak{J}_{j}= (N-1 )\varGamma-\varDelta \quad \forall i, $$

since we have used the identity $\sum_{j=1}^{F-1}\cos (\frac{2\pi}{F}j )=-1$. So this connectivity satisfies the condition introduced above, with $\mathcal{R}=\frac{\mathscr{A}' (\mu )}{N-1}$, where for symmetry reasons *μ* is the solution of equation $\mu=\tau [\frac{\mathcal{S}}{N-1}\mathscr{A} (\mu )+\mathcal {C} ]$ (so for $\varDelta =0$ we re-obtain Eq. (6.1), as it must be). Moreover, the eigenquantities of $T\circ\overline{J}^{c}$ are known, since this is a block-circulant matrix, therefore those of $\mathcal{J}$ can be obtained straightforwardly.

Finally, this neural network can be extended to the case of multiple populations with different sizes and vertex degrees (of which a very special example is the complete *k*-partite graph, whose topology is generally irregular). The analysis is beyond the purpose of this work and will be developed in upcoming articles.

## Numerical Comparison

In this section we show that our first-order perturbative expansion is in good agreement with the real behavior of the neural network obtained from the simulation of the system (2.1). These stochastic differential equations have been solved numerically $10\mbox{,}000$ times with the Euler–Maruyama scheme, and this collection of trials has been used to calculate the correlation by a Monte Carlo method (the code, running under Python 2.6, is available in the Supplementary Material). This result is then compared to the perturbative formula of the correlation obtained in the previous sections. The topologies that have been chosen for this comparison are $C_{10}$, $K_{10}$, $\mathcal{BC}_{3,10} (4,5,5 )$ and $Q_{4}$ (see Figs. 2 and 3), while the values of the parameters used in the numerical simulations are shown in Table 1. Moreover, the variable part of the synaptic weights and the external input currents have been chosen as follows:

7.1 $$ \begin{aligned} J_{ij}^{v} (t )&= \textstyle\begin{cases} \frac{1}{1+t^{2}}, & i,j=0,\ldots,\frac{N}{2}-1, \\ \frac{1}{2} [1+\operatorname{erf} (2t ) ], & i=0,\ldots,\frac{N}{2}-1, j=\frac{N}{2},\ldots,N-1, \\ \frac{1}{2} [1+e^{-t}\cos (3t ) ], & i=\frac {N}{2},\ldots,N-1, j=0,\ldots,\frac{N}{2}-1, \\ 1, & i,j=\frac{N}{2},\ldots,N-1. \end{cases}\displaystyle \\ I_{i}^{v} (t )&= \textstyle\begin{cases} \sin (4t ), & i=0,\ldots,\frac{N}{2}-1, \\ 1-e^{-2t}, & i=\frac{N}{2},\ldots,N-1. \end{cases}\displaystyle \end{aligned} $$

**Fig. 5:**
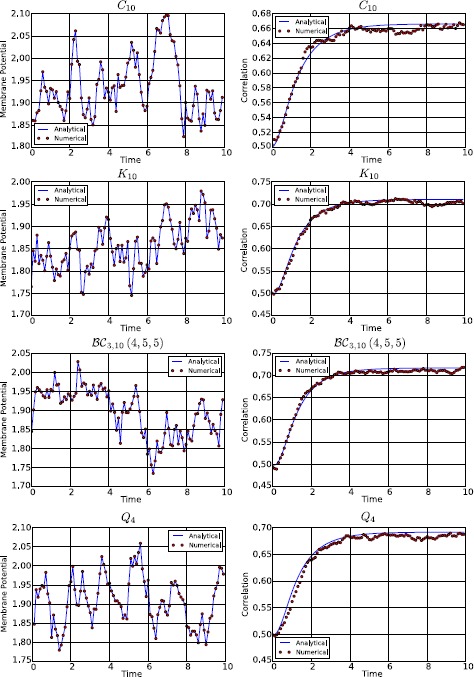
Comparison between the first-order perturbative expansion and the real behavior of the network, for the topologies $C_{10}$, $K_{10}$, $\mathcal {BC}_{3,10} (4,5,5 )$ and $Q_{4}$. The parameters used for the simulation are $\sigma=0.1$ and those shown in Table 1 and by Eq. (7.1). Correlation has been calculated by simulating equations in (2.1) $10\mbox{,}000$ times with the Euler–Maruyama scheme and then by applying a Monte Carlo method. Finally, this result is compared to the first-order analytical formula of the correlation. The figure shows good agreement, which validates the use of the perturbative approach

**Fig. 6:**
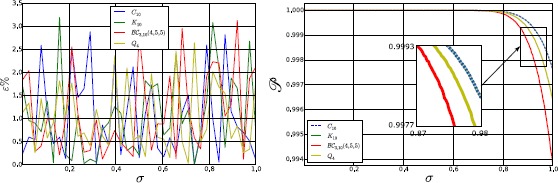
Percentage-relative error of the correlation calculated between the first-order perturbative expansion and the numerical simulation of the neural network (*left*) and the probability $\mathscr{P}$ defined by (4.13) (*right*), for $\sigma=10^{-3}-1$. The error is small (${<}3.5\%$) even for relatively large values of the perturbative parameter ($\sigma \thicksim1$), which proves the goodness of the perturbative approach. *ε*% increases considerably for $\sigma\gg1$, but this result has not been shown, since such values correspond to biologically unrealistic levels of randomness for a neural network. On the other hand, the figure shows that $\mathscr{P}\approx1$, which further confirms the legitimacy of the Taylor expansion (3.2) and therefore the validity of our results. Clearly $\mathscr{P}$ decreases with *σ* because a larger variance brings the membrane potential closer to the borders defined by the radius of convergence

We plot this comparison as a function of time (Fig. 5) and also the percentage-relative error

$$\varepsilon\%=100\times\biggl\vert \frac{\mathrm{numerical\ Corr}-\mathrm {fir{st}\ order\ perturbative\ Corr}}{\mathrm{numerical\ Corr}}\biggr\vert $$

as a function of the perturbative parameters (left-hand side of Fig. 6). In order to avoid high dimensional plots, we assume that $\sigma_{0}=\cdots=\sigma _{4}\overset{\mathrm{def}}{=}\sigma$.

**Table 1:** **Parameters used for the numerical simulations of Figs.** **5** **,** **6** **,** **7** **and the right-hand side of Fig.** **8** **. For the left-hand side of Fig.** **8** **and for Fig.** **9** **the parameters are the same, with only the exception of** $\pmb{C^{ (0 )}}$ **,** $\pmb{C^{ (1 )}}$ **and** $\pmb{C^{ (2 )}}$ **, which have been set to zero**

Neuron	Initial conditions	Synaptic weights	External input	Logistic function
*τ* = 1		*Γ* = 1	$I^{c}=1$	$\nu_{\mathrm{max}}=1$ *Λ* = 1 $V_{T}=0$
$C^{ (0 )}=0.4$	$C^{(1 )}=0.5$	$C^{(2)}=0.6$		

Figure 5 has been obtained for $\sigma=0.1$ and it clearly shows that the membrane potential follows very closely its numerical counterpart, while for the correlation the difference between the numerical simulation and the perturbative formula is of order 10^−2^. This is compatible with the law of large numbers, according to which the statistical error introduced by a Monte Carlo method with $\mathcal{T}$ trials is of order $\sqrt{\mathcal{T}}$.

The error *ε*% has been calculated as a function of the perturbative parameter, for $\sigma=10^{-3}-1$. Since we want to take into account also the error introduced by the perturbative expansion with respect to the initial conditions, whose effect quickly vanishes due to the time constant *τ*, the error *ε*% has been calculated at a small time instant, namely $t=1$. The result is shown in the left-hand side of Fig. 6, which confirms the goodness of the perturbative approximation, since the error is always smaller than 3.5% if calculated over $10\mbox{,}000$ trials. *ε*% could be even smaller if $\mathcal{T}$ is increased.

The right-hand side of Fig. 6 shows the numerical evaluation of the probability $\mathscr{P} (t )$ for $t=1$ (see (4.13)) according to the algorithm introduced in [49]. From the figure it is easy to check that for $\sigma=10^{-3}-1$ we obtain $\mathscr {P}\approx1$, which further confirms the validity of our results.

**Fig. 7:**
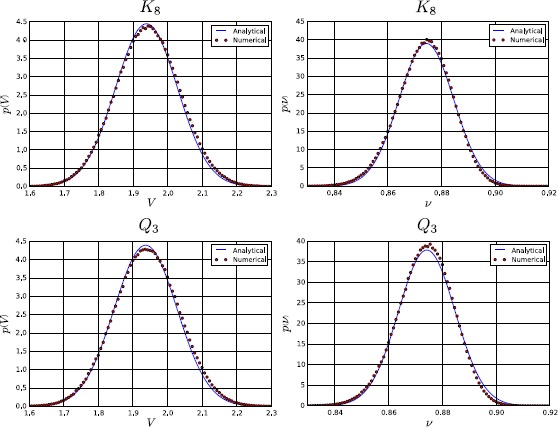
Single-neuron marginal-probability density for the membrane potential (*left*) and the firing rate (*right*) in a network with topology $K_{8}$ (*top*) and $Q_{3}$ (*bottom*). The parameters used for the simulation are $t=1$, $\sigma=0.1$, and those of Table 1 and Eq. (7.1). The numerical probability density has been calculated by simulating equations in (2.1) $1\mbox{,}000\mbox{,}000$ times with the Euler–Maruyama scheme and then by applying a Monte Carlo method, while the analytical density has been evaluated by integrating Eqs. (4.11) + (4.12) over all but one dimension. From the comparison it is easy to observe that the mean and the variance of the numerical simulations are in good agreement with the corresponding analytical quantities, even if the numerical probability density is not perfectly normal, due to relatively small higher-order corrections that have been neglected in our first-order perturbative approach

To conclude, in Fig. 7 we show a comparison between the numerical and analytical probability density for both the membrane potential and the firing rate, in networks with topologies $K_{8}$ and $Q_{3}$. Again, the parameters used in the simulations are $t=1$, $\sigma=0.1$, and those of Table 1 and Eq. (7.1). For the sake of clarity we have considered only the single-neuron marginal probability, since it facilitates the comparison. The numerical probability has been calculated by solving the system (2.1) $1\mbox{,}000\mbox{,}000$ times and by applying a Monte Carlo method, while the analytical density has been evaluated by integrating Eqs. (4.11) + (4.12) over all but one dimension. The figure confirms that at the first order the neural network can be described by a normal process, even if small deviations from the normal distribution, due to the non-linearity introduced by $\mathscr{A} (V )$, can be observed.

## Correlation as a Function of the Strength of the Network’s Input

In this section we consider how the cross-correlation among neurons depends upon a crucial network parameter, namely the strength of the external input current $I^{c}$. As explained above, $I^{c}$ represents the external input to the network (for example, a feed-forward input from the sensory periphery, or a top-down modulatory input) that drives or inhibits the activity of our network. Studying how the network properties depend on the parameter $I^{c}$ is important for many reasons. From the mathematical and theoretical point of view, this is important because this parameter may profoundly affect network dynamics. For example, the input can change the dynamical behavior of the system from a stationary to an oscillatory activity, because the eigenvalues of the Jacobian matrix (3.9) depend on *μ*, which in turn is determined by $I^{c}$ through Eq. (6.1). So changing $I^{c}$ can transform real eigenvalues into imaginary ones (in non-symmetric connectivity matrices) and therefore generate oscillations, or change the sign of the real part of an eigenvalue from negative to positive, giving rise to an instability. From the neural coding point of view, characterizing the dependence of different aspects of network activity upon the external input is necessary to understand and quantify how different aspects of network activity take part in the encoding of external stimuli [50–53]. Here we investigate specifically how the correlations among neurons depend on $I^{c}$.

**Fig. 8:**
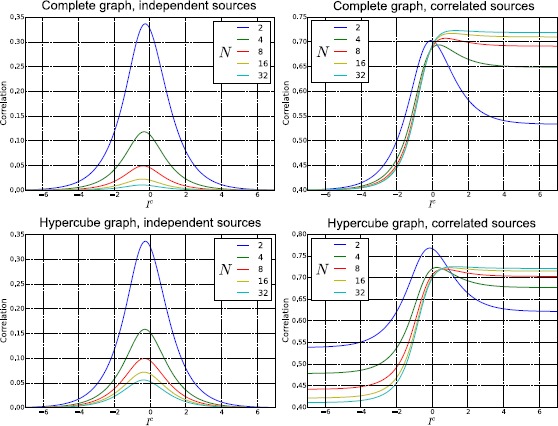
Correlation at $t=10$ and for $\sigma=0.1$, as a function of the external input current $I^{c}$, in the case of the complete (*top*) and hypercube (*bottom*) graph. The figure has been obtained for both independent (*left*) and correlated (*right*) sources of randomness. In a more detail, for the correlated case these results have been obtained with the parameters of Table 1, while for the independent case the parameters are the same, with only the exception of $C^{ (0 )}$, $C^{ (1 )}$, and $C^{ (2 )}$, which have been set to zero. This figure shows that the correlation is strongly modulated by $I^{c}$, confirming its relation with the effective connectivity matrix $J^{\mathrm{eff}}$

The dependence of correlation on $I^{c}$ is shown in Fig. 8. In this figure, the top panels show correlations for any pair of neurons in a network with a complete connectivity graph (in which case, the correlation has the same value for all pairs of neurons and so is independent of the neural indices *i*, *j*). The bottom panels show the correlation values for a pair of directly connected neurons in a hypercube graph (in this network, the correlation value depends only on the distance between two vertices, i.e. the number of edges in a shortest path connecting them, which can range between the value of 1 which corresponds to directly connected vertices, and the maximal value of log_2_*N*).

We first examined the case when the sources of variability are independent (left panels of Fig. 8), i.e. when $C^{ (0 )}$, $C^{ (1 )}$, and $C^{ (2 )}$ are equal to zero. Considering (3.10), it is apparent that this behavior originates from the sigmoidal shape of the activation function: when $\vert I^{c}\vert $ is large, then $\vert \mu \vert $ is large as well, therefore $\mathscr{A}' (\mu )$ and the entries of the effective connectivity matrix are small. In other words, the neurons become effectively disconnected, due to the saturation of the sigmoidal activation function. An important consequence of this phenomenon is that the neurons become independent, even if the size of the network is finite. This result holds for both the complete (top-left panel) and the hypercube graph (bottom-left panel of Fig. 8). An important implication of this result is that, taking into account that *ν* increases with $I^{c}$, in general $\operatorname{Corr} (\nu_{i} (t ),\nu_{j} (t ) )$ is not a monotonic function of the firing rate.

When the sources of variability are correlated, we found (for both network topologies; see right panels of Fig. 8) that the dependence of the correlation upon the parameter $I^{c}$ was very different from the case of uncorrelated sources of variability. In this case, for both considered topologies, $\operatorname {Corr} (\nu _{i} (t ),\nu_{j} (t ) )$ increases with the firing rate provided that the sources of randomness were sufficiently correlated and the network is large enough (see the case $N=32$ in the right panels of Fig. 8).

## Failure of Sznitman’s Mean-Field Theory

In this section we take advantage of our ability to study generic networks to investigate the ranges of applicability of Sznitman’s mean-field theory for the mathematical analysis of a neural network. A neural network is generally described by a large set of stochastic differential equations, which makes it hard to understand the underlying behavior of the system. However, if the neurons become independent, their dynamics can be described with the mean-field theory using a highly reduced set of equations that are much simpler to analyze. For this reason the mean-field theory is a powerful tool that can be used to understand the network. One of the mechanisms through which the independence of the neurons can be obtained is the phenomenon known as *propagation of chaos* [19–22]. Propagation of chaos refers to the fact that, if we choose chaotic initial conditions for the membrane potentials, then any fixed number of neurons are independent $\forall t>0$ in the so called *thermodynamic limit*, namely when the number of neurons in the system grows to infinity. Therefore the term *propagation* refers to the “transfer” of the chaotic distribution of the membrane potentials from $t=0$ to $t>0$. Under simplified assumptions as regards the nature of the network (namely that the other sources of randomness in the system, in our case the Brownian motions and the synaptic weights, are independent), propagation of chaos does occur. However, in Sects. 9.1, 9.2 and 10 we show that in many cases of practical interest, e.g. for a system with either correlated Brownian motions, initial conditions and synaptic weights, or with a sufficiently sparse connectivity matrix, or with an arbitrarily large (but still finite) size, the correlation between pairs of neurons can be high. Therefore in general any fixed number of neurons are not independent, which invalidates the use of Sznitman’s mean-field theory for analyzing such networks.

### Chaos Does not Occur if the Sources of Randomness Are not Independent

Here the proof is provided through a simple counterexample, namely the complete graph. From (6.15) we obtain, in the limit $N\rightarrow\infty$:

$$\begin{aligned} &\operatorname{Corr} \bigl(V_{i} (t ),V_{j} (t ) \bigr) \\ &\quad= \biggl(\sigma_{0}^{2}C^{ (0 )}\frac{e^{2\widetilde {\lambda}_{0}t}-1}{2\widetilde{\lambda}_{0}}+\sigma_{1}^{2}C^{ (1 )}e^{2\widetilde{\lambda}_{0}t}+\sigma_{2}^{2}C^{ (2 )}\mathscr{A}^{2} (\mu ) \biggl(\frac {e^{\widetilde{\lambda}_{0}t}-1}{\widetilde{\lambda}_{0}} \biggr)^{2}\biggr) \\ &\qquad{}\bigg/\biggl(\sigma_{0}^{2} \biggl[C^{ (0 )}\frac{e^{2\widetilde {\lambda}_{0}t}-1}{2\widetilde{\lambda}_{0}}-\frac{\tau}{2} \bigl(1-C^{ (0 )} \bigr) \bigl(e^{-{2t}/{\tau}}-1 \bigr) \biggr] \\ &\qquad{}+\sigma_{1}^{2} \bigl[C^{ (1 )}e^{2\widetilde {\lambda}_{0}t}+ \bigl(1-C^{ (1 )} \bigr)e^{-{2t}/{\tau}} \bigr]+\sigma_{2}^{2}C^{ (2 )}\mathscr {A}^{2} (\mu ) \biggl(\frac{e^{\widetilde{\lambda }_{0}t}-1}{\widetilde{\lambda}_{0}} \biggr)^{2}\biggr) . \end{aligned}$$

From this formula it is easy to see that if at least one of the parameters $C^{ (0 )}$, $C^{ (1 )}$, and $C^{ (2 )}$ is not equal to zero, then $\operatorname {Corr} (V_{i} (t ),V_{j} (t ) )\neq0$ (absence of chaos), even if we are in the thermodynamic limit. In particular, this means that:

- if $C^{ (0 )},C^{ (2 )}\neq0$, then $C^{ (1 )}=0$ does not imply $\operatorname{Corr} (V_{i} (t ),V_{j} (t ) )=0$ (i.e. there is no propagation of initial chaos);
- at every finite *t*, if $C^{ (1 )}\neq0$, then $C^{ (0 )},C^{ (2 )}=0$ does not imply $\operatorname{Corr} (V_{i} (t ), V_{j} (t ) )=0$ (i.e. absence of initial chaos does not lead to chaos).

Therefore $\operatorname{Corr} (V_{i} (t ),V_{j} (t ) )=0$ can be obtained only for $C^{ (0 )}=C^{ (1 )}=C^{ (2 )}=0$, which is compatible with Sznitman’s mean-field theory. However, in the next section we will see that even under the last condition, namely even if all the sources of randomness are independent, propagation of chaos may not occur if the neurons are not densely connected. Clearly the fully connected network has the largest number of connections possible, for this reason it does show propagation of chaos in the thermodynamic limit. Other topologies may not satisfy this requirement.

### Propagation of Chaos Does not Occur in Sufficiently Sparse Networks

Again, we show this through a counterexample. Since in this section we are interested in sparse systems, we study propagation of chaos in the thermodynamic limit as a function of the number of connections in a circulant and block-circulant network. To this purpose, we set $C^{ (0 )}=C^{ (1 )}=C^{ (2 )}=0$ (see previous section). For $N\rightarrow\infty$ and finite *M*, the right-hand sides of equations in (6.7) do not converge to zero, therefore for every finite value of *M* propagation of chaos does not occur.

**Fig. 9:**
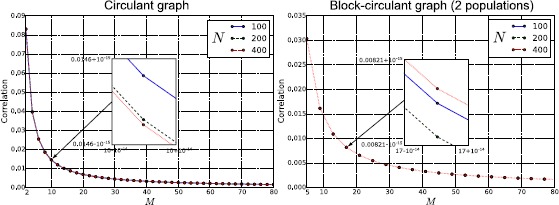
Correlation at $t=10$ and for $\sigma=0.1$, as a function of the number of incoming connections *M*, in the case of the circulant connectivity matrix $\operatorname{Ci}_{N} (1,2,\ldots,\xi )$ (*left*) and of the block-circulant matrix $\mathcal{BC}_{2,{N}/{2}} (\mathcal{M}_{0},\mathcal {M}_{1} )$, with $\mathcal{M}_{0}=\mathcal{M}_{1}-1=2\xi-H (\xi-\frac {N}{4}+1 )$ (*right*). The number of incoming connections is, respectively, $M=2\xi -H (\xi-\frac{N}{2}+1 )$ with $1\leq\xi\leq \lfloor\frac{N}{2} \rfloor$, and $M=1+4\xi-2H (\xi-\frac{N}{4}+1 )$ with $1\leq\xi\leq \lfloor\frac{N}{4} \rfloor$. These results have been obtained by using Eq. (6.7) with $C^{ (0 )}=C^{ (1 )}=C^{ (2 )}=0$ (while all the remaining parameters are those of Table 1). The figure shows that correlation does not go to zero in the thermodynamic limit (absence of propagation of chaos) if $\lim_{N\rightarrow \infty}M$ is finite, namely if the network is sufficiently sparse

However, from Fig. 9 we see that correlation decreases with *M*, therefore propagation of chaos occurs only in the thermodynamic limit and if *M* is an increasing function of *N*, namely if $\lim_{N\rightarrow\infty }M=\infty$. For example, in the complete graph $M=N-1$, so it explains why in this case correlation goes to zero in the thermodynamic limit. Instead in a network with a cyclic topology, propagation of chaos is never possible, also for $N\rightarrow\infty$, since $M=2$. In other words, having infinitely many neurons is not a sufficient condition for getting independence, because also infinite connections per neuron are required.

## Stochastic Synchronization

Finally, we use our formalism to demonstrate a theoretically interesting regime of network dynamics. In particular, we show that for every finite and arbitrarily large number of neurons in the network, it is possible to choose special values of the parameters of the system such that, at some finite and arbitrarily large time instant, correlation is (approximately) equal to one. In other terms, the stochastic components of the membrane potentials become perfectly synchronized, therefore from now on we refer to this phenomenon as *stochastic synchronization*. This is a very counterintuitive behavior of the network, since it does occur even when all the sources of randomness are independent (namely $C^{ (0 )}=C^{ (1 )}=C^{ (2 )}=0$). It is important to observe that this phenomenon requires a precise tuning of the parameters of the network, which is really hard to find by chance through numerical simulations. For this reason we need a rigorous theory that tells us how to set the parameters: such a theory is developed in the next section.

### The General Theory

More precisely, here we show that even when $C^{ (0 )}=C^{ (1 )}=C^{ (2 )}=0$, if the Jacobian matrix (3.9) has an eigenvalue of algebraic multiplicity one with non-negative real part, while all the other eigenvalues have negative real parts, then correlation goes to one for $t\rightarrow\infty$, for every finite *N*. This is proved for a generic anatomical connectivity, therefore the assumption of regularity is relaxed. To prove this result, we suppose that $\mathcal{J}$ has an eigenvalue $\widetilde{\lambda}_{\mathrm{max}}$ with non-negative real part and with a generic algebraic multiplicity $m>0$, while all the other eigenvalues have negative real parts. Now from (3.11) we recall that $D (t )$ is the diagonal matrix of the eigenvalues of $e^{\mathcal{J}t}$, and *P* is the matrix of its eigenvectors. If $\widetilde{\lambda}_{\mathrm{max}}$s are the first *m* eigenvalues of $\mathcal{J}$, for $t\rightarrow\infty$ we have 
 because all the eigenvalues have negative real part but $\widetilde{\lambda}_{\mathrm{max}}$. Therefore

$$\begin{aligned} PD (t )P^{-1}\approx{}& e^{\widetilde{\lambda}_{\mathrm {max}}t} \left[ \textstyle\begin{array}{@{}c@{\quad}c@{\quad}c@{\quad}c@{\quad}c@{\quad}c@{}} P_{0,0} & \cdots& P_{0,m-1} & 0 & \cdots& 0 \\ P_{1,0} & \cdots& P_{1,m-1} & 0 & \cdots& 0 \\ \vdots& \ddots& \vdots& \vdots& \ddots& \vdots \\ P_{N-1,0} & \cdots& P_{N-1,m-1} & 0 & \cdots& 0 \end{array}\displaystyle \right] \\ &{}\times\left[ \textstyle\begin{array}{@{}c@{\quad}c@{\quad}c@{}} [P^{-1} ]_{0,0} & \ldots& [P^{-1} ]_{0,N-1} \\ {} [P^{-1} ]_{1,0} & \ldots& [P^{-1} ]_{1,N-1} \\ \vdots& \ddots& \vdots \\ {} [P^{-1} ]_{N-1,0} & \ldots& [P^{-1} ]_{N-1,N-1} \end{array}\displaystyle \right] \end{aligned}$$

and, moreover,

$$\begin{aligned} e^{\mathcal{J}t} \approx& e^{\widetilde{\lambda}_{\mathrm {max}}t}E, \\ E_{pq} = & \sum_{l=0}^{m-1}P_{pl} \bigl[P^{-1} \bigr]_{lq}. \end{aligned}$$

According to Eqs. (4.4)–(4.6) for $C^{ (0 )}=C^{ (1 )}=C^{ (2 )}=0$, this means that

$$\begin{aligned} &\operatorname{Cov} \bigl(V_{i} (t ),V_{j} (t ) \bigr) \\ &\quad= \sigma_{0}^{2} \sum_{k=0}^{N-1} \int_{0}^{t} \bigl[e^{\mathcal {J} (t-s )} \bigr]_{ik} \bigl[e^{\mathcal{J} (t-s )} \bigr]_{jk}\,ds+ \sigma_{1}^{2} \sum_{k=0}^{N-1} \bigl[e^{\mathcal{J}t} \bigr]_{ik} \bigl[e^{\mathcal{J}t} \bigr]_{jk} \\ &\qquad{} +\sigma_{2}^{2}\frac{\mathscr{A}^{2} (\mu )}{M} \sum _{k=0}^{N-1} \biggl(\int_{0}^{t} \bigl[e^{\mathcal{J} (t-s )} \bigr]_{ik}\,ds \biggr) \biggl(\int _{0}^{t} \bigl[e^{\mathcal {J} (t-s )} \bigr]_{jk}\,ds \biggr) \\ &\quad \approx \biggl(\sigma_{0}^{2}\frac{e^{2\widetilde{\lambda }_{\mathrm{max}}t}-1}{2\widetilde{\lambda}_{\mathrm{max}}}+\sigma _{1}^{2}e^{2\widetilde{\lambda}_{\mathrm{max}}t}+\sigma _{2}^{2} \frac{\mathscr{A}^{2} (\mu )}{M} \biggl(\frac {e^{\widetilde{\lambda}_{\mathrm{max}}t}-1}{\widetilde{\lambda }_{\mathrm{max}}} \biggr)^{2} \biggr) \sum _{k=0}^{N-1} E_{ik}E_{jk} \end{aligned}$$

where for $\widetilde{\lambda}_{\mathrm{max}}=0$ we mean $\frac{e^{\gamma\widetilde{\lambda}_{\mathrm{max}}t}-1}{\gamma \widetilde{\lambda}_{\mathrm{max}}}=t$, given $\gamma\in \{ 1,2 \} $. Therefore

$$\lim_{t\rightarrow\infty}\operatorname{Corr} \bigl(V_{i} (t ),V_{j} (t ) \bigr)=\frac{ \sum_{k=0}^{N-1} E_{ik}E_{jk}}{\sqrt{ [ \sum_{k=0}^{N-1} (E_{ik} )^{2} ] [ \sum_{k=0}^{N-1} (E_{jk} )^{2} ]}}. $$

Now, in the special case $m=1$ we obtain

$$\begin{aligned} E_{pq}&=P_{p0} \bigl[P^{-1} \bigr]_{0q}, \\ \sum_{k=0}^{N-1}E_{ik}E_{jk}&= \sqrt{ \Biggl[ \sum_{k=0}^{N-1} (E_{ik} )^{2} \Biggr] \Biggl[ \sum _{k=0}^{N-1} (E_{jk} )^{2} \Biggr]}=P_{i0}P_{j0}\sum_{k=0}^{N-1} \bigl( \bigl[P^{-1} \bigr]_{0k} \bigr)^{2} , \end{aligned}$$

so we conclude that $\lim_{t\rightarrow\infty}\operatorname {Corr} (V_{i} (t ),V_{j} (t ) )=1$. In other terms, the neurons become perfectly correlated even if the sources of randomness are independent, which is what we wanted to prove.

It is interesting to observe that, due to the Perron–Frobenius theorem [54], if the matrix with entries $\frac {1}{M_{i}}J_{ij}^{\mathrm{eff}}$ (see Eq. (3.9)) is non-negative and irreducible (namely if its corresponding directed graph is *strongly connected*, which means that it is possible to reach each vertex in the graph from any other vertex, by moving on the edges according to their connectivity directions), then it has a unique largest positive eigenvalue, which can be used to generate stochastic synchronization.

To conclude, it is important to observe that we must be careful when we use the perturbative expansion to describe stochastic synchronization. Actually the divergence of the term $e^{\gamma\widetilde{\lambda }_{\mathrm{max}}t}$ implies a fast growth of the variance of the membrane potential, therefore the first-order approximation may not be good enough due to a possibly larger magnitude of the higher-order perturbative corrections. However, this problem can easily be fixed by choosing sufficiently small values of $\sigma_{m}$ that ensure the variance is still small when the correlation is close to one. Another possibility is to choose the parameters of the network in order to have $\widetilde{\lambda }_{\mathrm{max}}$ negative but very close to zero. For continuity, in this case correlation will be very close to one, and the variance cannot diverge since $\widetilde{\lambda}_{\mathrm{max}}<0$.

Now we are ready to see an explicit example of stochastic synchronization, which will be developed in the next section for the complete and the hypercube graphs.

### Examples: The Complete and the Hypercube Graphs

For both these topologies, the largest eigenvalue is $\widetilde {\lambda}_{\mathrm{max}}=-\frac{1}{\tau}+\varGamma\mathscr{A}' (\mu )$ with algebraic multiplicity one. According to Sect. 10.1, we have to set $\widetilde{\lambda}_{\mathrm{max}}\geq0$ in order to obtain stochastic synchronization. In particular, we consider the case $\widetilde{\lambda}_{\mathrm{max}}=0$ and we use the logistic function $\mathscr{A} (V )=X (V )$, since we can take advantage of the following property:

$$X' (\mu )=\varLambda \biggl[X (\mu )-\frac {X^{2} (\mu )}{\nu_{\mathrm{max}}} \biggr]. $$

Now, the condition $\widetilde{\lambda}_{\mathrm{max}}=0$ can be rewritten as $\varGamma X' (\mu )=\frac{1}{\tau}$, namely

$$\varGamma\varLambda \biggl[X (\mu )-\frac{X^{2} (\mu )}{\nu_{\mathrm{max}}} \biggr]=\frac{1}{\tau}. $$

The solutions of this algebraic equation are

10.1 $$ X (\mu_{1,2} )=\nu_{\mathrm{max}}\frac{1\pm\sqrt {1-{4}/{(\tau\varGamma\varLambda\nu_{\mathrm{max}})}}}{2} $$

where $\mu_{1,2}$ are two possible stationary solutions of the membrane potential. Moreover, from Eq. (6.1) we know that

10.2 $$ \mu_{1,2}=\tau \bigl[\varGamma X (\mu_{1,2} )+I^{c} \bigr]. $$

Putting together Eqs. (10.1) and (10.2) we obtain

$$\mu_{1,2}=\tau \biggl(\varGamma\nu_{\mathrm{max}}\frac{1\pm\sqrt {1-{4}/{(\tau\varGamma\varLambda\nu_{\mathrm {max}})}}}{2}+I^{c} \biggr) . $$

Replace this value of $\mu_{1,2}$ in (10.2) to obtain the final result:

$$\nu_{\mathrm{max}}\frac{1\pm\sqrt{1-{4}/{(\tau\varGamma\varLambda \nu_{\mathrm{max}})}}}{2}=X \biggl(\tau \biggl(\varGamma \nu_{\mathrm {max}}\frac{1\pm\sqrt{1-{4}/{(\tau\varGamma\varLambda\nu_{\mathrm {max}})}}}{2}+I^{c} \biggr) \biggr). $$

This non-linear algebraic equation is the constraint that must be satisfied by all the parameters of the system in order to have correlation equal to 1 in the limit $t\rightarrow\infty$. An example of solution of this equation is

10.3 $$ \begin{aligned} \varLambda&=\nu_{\mathrm{max}}=1,\qquad V_{T}=0, \\ \varGamma&=-2I^{c},\qquad\tau=-\frac{2}{I^{c}},\quad\forall I^{c}< 0. \end{aligned} $$

In this case $\mu_{1,2}=0$ and it should be used as initial condition in order to ensure the stationarity of the system.

**Fig. 10:**
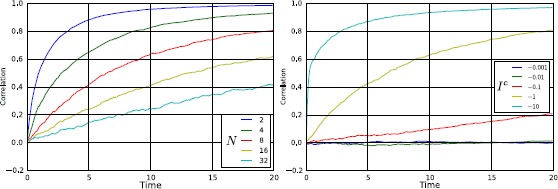
Stochastic synchronization in a fully connected network. These results have been obtained with the exact non-linear equations in (2.1) for $10\mbox{,}000$ trials. The parameters used in the simulation have been chosen according to the constraint (10.3), while for the sources of randomness we have $\sigma=0.01$ and $C^{ (0 )}=C^{ (1 )}=C^{ (2 )}=0$. In the example with $I^{c}=-1$, the figure shows that correlation gets closer and closer to one with a speed that depends on the number of neurons *N* in the system (*left*), and also stochastic synchronization as a function of $I^{c}$ in a network with $N=8$ (*right*)

In Fig. 10 we show the phenomenon of stochastic synchronization only in the case of the complete graph (for the hypercube the results are qualitatively similar). As we can see, correlation goes to one more and more slowly if we increase the number of neurons *N* in the network or if we decrease the current $I^{c}$. Therefore in the limit $N\rightarrow\infty$ and/or $I^{c}\rightarrow0$ the system has correlation 0 at every finite time instant. Actually, from (6.15) it is possible to prove that, given $t\gg1$, the time instant $t^{*}$ such that $\operatorname{Corr} (V_{i} (t^{*} ),V_{j} (t^{*} ) )=C$ is

$$\begin{aligned} t^{*} \approx& -\frac{1}{2\widetilde{\lambda}_{1}}\frac{1+C (N-1 )}{1-C}, \\ \widetilde{\lambda}_{1} =& -\frac{1}{\tau}-\frac{\varGamma\mathscr {A}' (\mu )}{N-1}= \frac{I^{c}}{2 (1-{1}/{N} )}, \end{aligned}$$

having used the fact that $\varGamma\mathscr{A}' (\mu )=\frac{1}{\tau}$ and $\tau=-\frac{2}{I^{c}}$. From this result we see that, for *C* fixed, $t^{*}$ increases linearly with *N* for large networks and is inversely proportional to $I^{c}$, as obtained numerically in Fig. 10. In particular, this proves that in the thermodynamic limit there is still propagation of chaos at every finite time instant. This is in agreement with Sznitman’s mean-field theory and the results on propagation of chaos proved in [20–22].

Moreover, from (B.1), it is interesting to observe that if there is a perfect stochastic synchronization between pairs of neurons, then it is “transmitted” to all the higher-order correlations with even order, at least for the complete graph. In other terms, if the neurons are all-to-all connected, then $\operatorname{Corr}_{2} (V_{i} (t ),V_{j} (t ) )=1$ implies $\operatorname{Corr} _{n} (V_{i_{0}} (t ),\ldots ,V_{i_{n-1}} (t ) )=1$, ∀*n* even.

## Discussion

In this article we developed a novel formalism for evaluating analytically the cross-correlation structure of a finite-size firing-rate network with recurrent connections, using a first-order perturbative expansion of the neural equations. Importantly, the network we considered is stochastic and includes three distinct sources of randomness, namely the background noise of the membrane potentials, their initial conditions and the distribution of the recurrent synaptic weights. With this approach we succeeded in calculating analytically correlations at any order among all groups of neurons in the network. This formalism is general and in principle can be applied to networks with any kind of topology of the anatomical connections, but here we applied it to the case of regular graphs. In upcoming articles this technique will be employed to study more general kinds of anatomical connections. In other terms, the present article represents a proof of concept of the ability of our theory to relate analytically the anatomical and functional connectivity.

The cases we have decided to study are networks with block-circulant and hypercube topologies. Clearly some of the results we have obtained could be specific for these special graphs. Nevertheless, our formalism applied to these cases has shown a series of (to our knowledge) new results, whose generality or specificity can be later determined by comparison with other kinds of anatomical connections.

### Dependence of the Correlation Structure on the Parameters of the System

First of all we quantified analytically how the correlation depends dynamically on the external input of the network. This has revealed a number of new and partly counterintuitive insights. We have shown that a strong input can make the neurons almost independent, and this reveals a simple mechanism to achieve network decorrelation that adds to those, such as the balance of excitation and inhibition (e.g. [27, 55]) or the use of purely inhibitory feedback (e.g. [56]), that were recently proposed. Moreover, we have shown that it is not possible to obtain a mean-field description à la Sznitman of the neural network, if the anatomical connections are too sparse or our three sources of variability are correlated. We have also proved that correlation depends not only on the input, but also on the topology of the network and on the correlation structure of the sources of randomness. To conclude, we have shown that for very special values of the parameters, the neurons become almost perfectly correlated even if the sources of randomness are independent. We have called this phenomenon *stochastic synchronization*, and we stress the fact that the formalism developed in this article is able to prove its existence for a completely generic anatomical connectivity whose eigenvalues satisfy a bland condition.

The dependence of network correlations on the neuron’s firing rates has been the subject of extensive investigations in recent years [57–59]. Our study of the dependence of the correlation on the strength of the external input allowed us to consider analytically this problem in our network. It is interesting to compare our results to those obtained in [57] for in-vitro real networks and for model integrate-and-fire networks. They reported that $\operatorname{Corr} (\nu_{i} (t ),\nu_{j} (t ) )$ increases with the geometric mean of the firing rates. However, in our model, this is not always the case. This happened in our case for strongly correlated inputs and relatively large networks (a scenario compatible with the cases studied in [57]). However, in our model the network showed a non-monotonic dependence of the correlation on the firing rates in other instances. A consequence of this non-monotonic dependence is that rates and correlations expressed by recurrent networks can indeed act as separate information channels for the encoding of the strength of the external stimuli. We would also like to underline the fact that, according to those authors, the correlation between the firing rates is bounded by the correlation between the inputs. According to our model, this is generally correct, but in some cases the neural network is able to generate almost perfectly correlated firing rates even if the inputs are independent. This is the phenomenon of *stochastic synchronization* discussed in Sect. 10.

### Strengths and Weaknesses of the Presented Approach

As discussed in Sect. 1, our approach presents some advantages when compared to other methods based on linear response theory [23–25], networks of stochastic binary neurons [26, 27], the linear noise approximation [28], the density functional approach [29], and large deviations theory [30–32]. These advantages consist in the possibility to use different sources of variability, to study synchronization and the effect of axonic delays, and to quantify finite-size effects also for small-size networks. This means that our formalism lends itself to the possibility of multiple generalizations and extensions. Additional sources of stochasticity, such as a random threshold $V_{T}$ in the activation function or a stochastic membrane time constant *τ*, can be introduced in the model even including correlations among different sources. As we stated above, delays in the transmission of the electric signal through the axons can be taken into account as well, following [60, 61]. Another possibility of further extensions of this study is the introduction of Hebbian learning. In this article we assumed for simplicity that the dynamics of the synaptic weights is already known, through the functions (2.5). However, in the case of synaptic plasticity the time evolution of the matrix $J (t )$ depends on the membrane potentials $V (t )$, so the system of differential equations (2.1) should be extended to include the differential version of Hebb’s learning rule. We also observe that in this article we have considered a deterministic topology *T* for the anatomical connectivity, which means that *T* is fixed from trial to trial. An interesting extension is the study of random topologies, in particular random regular graphs [62], but this problem will be tackled in another article.

A detailed analysis of the limits of our formalism for different values of all the parameters of the model and many graph topologies is beyond the purpose of the article. Nevertheless, being a perturbative approach, in general it is possible to assert that our method presents the same limits and advantages elucidated by (non-singular) perturbation theory, to which the interested readers are referred. Our formalism can be applied also to other neural equations, such as the Wilson–Cowan model [63]. However, it is important to observe that it requires the existence of a stable equilibrium point, around which the neural equations are linearized. Therefore this technique cannot be used to study the correlation structure of spiking neurons, like those described by FitzHugh–Nagumo [64, 65] or the Hodgkin–Huxley [66] or integrate-and-fire [67] neurons, because in these systems spikes are generated by periodic orbits. For example, for FitzHugh–Nagumo and Hodgkin–Huxley neurons, stable periodic orbits occur around unstable equilibria, therefore our method predicts the divergence of the covariance matrix for $t\rightarrow\infty$, which is clearly a consequence of the linearization of the neural equations. This also means that our formalism cannot be used to evaluate the correlation structure when equations (2.1) undergo neural oscillations generated through Hopf bifurcations, but can still describe damped oscillations around a stable focus in the phase space when the connectivity matrix has complex eigenvalues.

Another difficulty of our formalism is the need for an analytical expression of the eigenquantities of the Jacobian matrix $\mathcal{J}$, of which we have shown a biologically relevant example in Sect. 6.3.2. Clearly spectra of brain areas that accomplish complex functions are difficult to evaluate analytically. For this reason we are forced to introduce some simplifications of the structural connectivity that we want to study. Another possibility is to determine the eigenquantities numerically, and then Eqs. (4.4)–(4.6) provide an algorithm for evaluating numerically the correlation structure of the network. Clearly even with this method the eigenquantities cannot be calculated for very large networks, since the matrix $\mathcal{J}$ is $N\times N$ and therefore grows quickly with the network size. However, the advantage of evaluating numerically Eqs. (4.4)–(4.6) is evident, compared to the Monte Carlo approach. Actually, if the randomness of the synaptic weights is taken into account (namely if $\sigma_{2}\neq0$), one needs to generate numerically by a random generator the $N^{2}$ entries of the matrix *W*, according to the covariance matrix (2.6), which has $N^{4}$ entries. This calculation must be repeated for a sufficiently high number of trials, according to the Monte Carlo method, so it is computationally much more expensive in terms of time and memory consumption.

It is important to observe that in this article we focused mainly on regular graphs for the sake of clarity, since for this class of connectivity matrices the eigenquantities of $\mathcal{J}$ can be evaluated easily from those of $T\circ\overline{J}^{c}$ through Eq. (6.2). For a general connectivity this relation is harder to find, but we underline that this is in part due to our choice to use a biologically realistic activation function $\mathscr{A} (\cdot )$ (see Eqs. (3.9) and (3.10)). Usually, in order to obtain analytical results, in the literature there is a wide use of piecewise linear activation functions (e.g. in [48, 68, 69]). Clearly in this case it is much easier to evaluate the eigenquantities of $\mathcal{J}$ from those of $T\circ\overline{J}^{c}$, taking some care at the connection points between the segments of $\mathscr {A} (\cdot )$, where the piecewise linear function is not differentiable.

Another useful feature of our approach is that it allowed the calculation of the dependence on the strength of the external input of correlations of arbitrary order (not only pairwise correlations). This feature will be useful for the evaluation of the ability of networks to encode genuinely additional information in the variations with inputs of higher-order correlations, a subject that has been under intense theoretical [70] and experimental debate in recent years [71, 72].

### Analyzing the Consequences of Structural Damage

Similarly to spectral graph theory, where the properties of a graph are studied in relationship to its characteristic polynomial and eigenquantities, in this article we have found the relation between the functional connectivity and the spectrum of the underlying structural connectivity. This, in principle, allows one to study the effect on the functional connectivity caused by lesions to the synaptic connections. These structural damages can be modeled as perturbations to the topology matrix. Thus, in principle they can be studied by perturbative techniques such as those described in [73–77]. This branch of graph theory deals with discrete perturbations (such as the removal of connections or vertices from a given graph), as opposed to the Rayleigh–Schrödinger theory from quantum mechanics, that studies the effect of continuous perturbations to the generalized eigenvalue problem. This approach would help to understand abnormal functional behavior, complementing other studies of the consequences of structural damage, e.g. [78].

### Possible Extensions to Other Measures of Communication Among Neurons

It is also interesting to observe that the correlation structure can be used to estimate causal relations between neurons or neural populations. This can be achieved in many ways. However, in our view a promising direction is to take advantage of hierarchical clustering techniques already used in economics, whose potential application is briefly described as follows. According to [79], the correlation structure can be used to define a distance measure $d_{ij} (t )\overset{\mathrm{def}}{=}\sqrt{2 (1-\operatorname {Corr} (V_{i} (t ),V_{j} (t ) ) )}$ between every pair of neurons. Clearly we are not interested in the hierarchical structure of single neurons, but rather in that of mesoscopic or macroscopic areas. For this reason, from $d_{ij} (t )$ we have to define an arbitrary distance between these areas of the brain (e.g. the mean distance between all the pairs of neurons). Then, from the distance matrix of the areas, we can determine the *minimum spanning tree* of the system, a concept introduced in the context of graph theory to find the most relevant (or more informative) connections in a network. Finally, on the minimum spanning tree it is possible to define an ultrametric distance, which in turn allows us to build a *dendrogram* (i.e. a hierarchical tree) in an unambiguous way, by using techniques such as *UPGMA* [80].

### Concluding Statement

We have shown that the formalism introduced in this article can be effectively used to calculate the functional connectivity of neurons within a firing-rate network model. In this article we concentrated mostly on computing the Pearson correlation among all pairs of neurons in the network. However, the work reported in this paper also lays the basis for computing more refined measures of functional connectivity (such as those based on information theory). This in turn will allow in future studies the analytical quantification of the transmission of information among the elements of this recurrent network and of how information transmission is modulated by factors such as the strength and dynamics of external inputs.

### Electronic Supplementary Material

Python code. (PY 24 KB)

## Figures and Tables

**Fig. 11:**
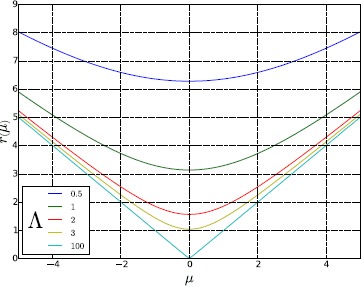
Radius of convergence *r* of the Taylor series of the logistic function $X (V )$, as a function of the point $V=\mu$ about which the expansion is performed. For large *μ* the radius of converge increases linearly since the logistic function is asymptotically flat. Instead for $\varLambda \rightarrow\infty$ we obtain $r (0 )\rightarrow0$, because in this limit the logistic function becomes a Heaviside step function with a discontinuity in $V=0$

## Appendix A: Radius of Convergence of Some Activation Functions

In this section we compute the radius of convergence of the logistic and inverse tangent function (see (2.2)). For simplicity we consider only the case with $\nu_{\mathrm{max}}=1$ and $V_{T}=0$, but the analysis can be extended easily to the most general case.

### A.1 The Logistic Function

According to [81], the *n*th-order derivative of the logistic function $X (V )$ is

A.1 $$ D^{n}X (V )=\varLambda^{n}\sum _{k=1}^{n} (-1 )^{k-1}A (n,k-1 ) \bigl[X (V ) \bigr]^{k} \bigl[1-X (V ) \bigr]^{n+1-k} $$

where $A (n,k )$ are the so called *Eulerian numbers* [82]. After some algebra, we can rewrite this expression as follows:

$$D^{n}X (V )=\varLambda^{n}X (V ) \bigl[1-X (V ) \bigr]^{n}\sum_{k=0}^{n-1}A (n,k ) \bigl(-e^{-\varLambda V} \bigr)^{-k}. $$

Now from [83], we know that

$$\operatorname{Li}_{-n} (V )=\frac{V^{n}}{ (1-V )^{n+1}} \sum _{k=0}^{n-1} A (n,k )V^{-k}, \quad \forall n>0, \vert V\vert < 1, $$

where $\operatorname{Li}_{-n} (\cdot )$ represents the so called *polylogarithm* (with negative order). Here we have omitted the *n*th term of the sum since $A (n,n )=0$$\forall n>0$. So we obtain

A.2 $$ D^{n}X (V )= (-\varLambda )^{n}\operatorname {Li}_{-n} \bigl(-e^{-\varLambda V} \bigr) . $$

This result is true as far as $\vert {-}e^{-\varLambda V}\vert <1$, i.e. only for $V>0$. Instead, for $V<0$, we can use the relation $X (-V )=1-X (V )$, from which we deduce that:

- $D^{n}X (-V )= (-1 )^{n-1}D^{n}X (V )$, $\forall n>0$;
- $X (-V )$ has the same radius of convergence of $X (V )$.

So Eq. (A.2) can be used to express $D^{n}X (V )$$\forall V\neq0$. Instead for $V=0$, according to (A.2), we have to evaluate $\operatorname{Li}_{-n} (-1 )$, which is defined by an analytical continuation of the polylogarithm. In this way we can determine $D^{n}X (0 )$. Another method is to use Eq. (A.1) and the following property of the Eulerian numbers:

$$\sum_{k=1}^{n} (-1 )^{k-1}A (n,k-1 )=2^{n+1} \bigl(2^{n+1}-1 \bigr)\frac{B_{n+1}}{n+1} $$

where $B_{n}$ are the so called *Bernoulli numbers* [84], from which we obtain

$$D^{n}X (0 )=\varLambda^{n} \bigl(2^{n+1}-1 \bigr) \frac{B_{n+1}}{n+1}. $$

Now we can compute the radius of convergence $r (\mu )$ of the Taylor series

$$X (V )=\sum_{n=0}^{\infty}\frac{D^{n}X (\mu )}{n!} (V-\mu )^{n} $$

using the Cauchy-root test:

A.3 $$ r (\mu )=\frac{1}{\limsup_{n\rightarrow\infty}\sqrt [n]{\vert {D^{n}X (\mu )}/{n!}\vert }}. $$

First of all we obtain $r (0 )=\frac{\pi}{\varLambda}$. This can be proved for example by performing the substitution $n\rightarrow2n-1$ (which is motivated by the fact that $B_{2n+1}=0$$\forall n>0$) and by using the following asymptotic expansion of the Bernoulli numbers:

$$B_{2n}\sim (-1 )^{n-1}4\sqrt{\pi n} \biggl( \frac{n}{\pi e} \biggr)^{2n}, \quad n\rightarrow\infty, $$

and the Stirling approximation of $(2n-1 )!$.

For $\mu\neq0$, from the relation between the polylogarithm and the *Hurwitz zeta function* [85], we recall the following result, valid for $n\rightarrow\infty$:

$$\operatorname{Li}_{-n} \bigl(-e^{-\varLambda\mu} \bigr)\sim n! \biggl[ \frac {1}{ (\varLambda\mu-\iota\pi )^{n+1}}+\frac{1}{ (\varLambda\mu+\iota\pi )^{n+1}} \biggr]; $$

therefore from (A.3) we obtain

$$r (\mu )=\frac{1}{ \mathop{\varLambda \limsup}_{n\rightarrow\infty}\sqrt[n]{\vert {1}/{ (\varLambda\mu-\iota\pi )^{n+1}}+{1}/{ (\varLambda\mu+\iota\pi )^{n+1}}\vert }}. $$

Now, since

$$\sqrt[n]{\biggl\vert \frac{1}{ (\varLambda\mu-\iota\pi )^{n+1}}+\frac{1}{ (\varLambda\mu+\iota\pi )^{n+1}}\biggr\vert }= \frac{1}{\rho^{1+{1}/{n}}}\sqrt[n]{\bigl\vert 2\cos \bigl( (n+1 )\theta \bigr)\bigr\vert } $$

where $\rho=\sqrt{ (\varLambda\mu )^{2}+\pi^{2}}$ and $\theta=\arctan (\frac{\pi}{\varLambda\mu} )$, and, moreover,

$$\lim_{n\rightarrow\infty}\sqrt[n]{\bigl\vert 2\cos \bigl( (n+1 )\theta \bigr)\bigr\vert }=1, $$

then we finally conclude that

$$r (\mu )=\frac{1}{\varLambda}\sqrt{ (\varLambda\mu )^{2}+ \pi^{2}}. $$

Figure 11 shows the result for different values of *Λ*. From it we can see that the radius of convergence of the Taylor series of $X (V )$ around the point $V=\mu$ increases with *μ*. This is reasonable, since the logistic function becomes flat when $\vert V\vert $ is large. Moreover, for large *Λ* it converges to $r (\mu )=\vert \mu \vert $ and therefore it is equal to zero only for $\mu=0$, as it must be. Actually, for $\varLambda\rightarrow\infty$ the function $X (V )$ converges to the Heaviside step function, which has a vertical jump at $V=0$.

### A.2 The Inverse Tangent Function

Now we calculate the radius of convergence of the inverse tangent function. According to [86], its *n*th-order derivative is

$$\begin{aligned} &D^{n} \biggl[\frac{1}{2}+\frac{1}{\pi}\arctan \biggl( \frac{\pi }{4}\varLambda V \biggr) \biggr] \\ &\quad=\frac{1}{\pi} \biggl( \frac{\pi }{4}\varLambda \biggr)^{n}\frac{ (-1 )^{n-1} (n-1 )!}{ [1+ (({\pi}/{4})\varLambda V )^{2} ]^{{n}/{2}}}\sin \biggl(n \arcsin \biggl(\frac{1}{\sqrt{1+ (({\pi}/{4})\varLambda V )^{2}}} \biggr) \biggr). \end{aligned}$$

So from the root test we obtain

$$r (\mu )=\frac{\sqrt{1+ (({\pi}/{4})\varLambda\mu )^{2}}}{({\pi}/{4})\varLambda\limsup_{n\rightarrow\infty }({\sqrt[n]{\vert \sin (n\arcsin ({1}/{\sqrt{1+ (({\pi}/{4})\varLambda\mu )^{2}}} ) )\vert }}/{\sqrt[n]{\pi n}})} . $$

Now, since

$$\lim_{n\rightarrow\infty}\sqrt[n]{\biggl\vert \sin \biggl(n\arcsin \biggl( \frac{1}{\sqrt{1+ (({\pi}/{4})\varLambda\mu )^{2}}} \biggr) \biggr)\biggr\vert }=1 $$

for *Λμ* finite, and, moreover, $\lim_{n\rightarrow\infty }\sqrt[n]{\pi n}=1$, we obtain finally

$$r (\mu )=\frac{1}{({\pi}/{4})\varLambda}\sqrt{1+ (({\pi}/{4})\varLambda\mu )^{2}}. $$

Therefore the radius of convergence increases with *μ*, as it must be. Moreover, in the limit $\varLambda\rightarrow\infty$ it gives $r (\mu )=\vert \mu \vert $, as with the logistic function. The same result can be proved for other sigmoidal functions and is left as an exercise for the reader.

## Appendix B: Higher-Order Correlations for a Fully Connected Neural Network

Here we calculate the higher-order correlation in the case of the complete graph. For this network topology, the combinatorial calculus required by the numerator of (4.1) is very simple:

$$\sum\prod\overline{\mathfrak{N}_{i_{j}} (t )\mathfrak {N}_{i_{k}} (t )}=\frac{n!}{2^{{n}/{2}} ({n}/{2} )!} \bigl(\overline{ \mathfrak{N}_{i} (t )\mathfrak{N}_{j} (t )} \bigr)^{{n}/{2}} $$

where the covariance $\overline{\mathfrak{N}_{i} (t )\mathfrak{N}_{j} (t )}$ is the same for every pair $(i,j )$ of neurons such that $i\neq j$, since they are all-to-all connected. For the denominator, Eq. (4.9) gives

$$\sqrt[n]{ \prod_{j=0}^{n-1} \overline{\bigl\vert \mathfrak {N}_{i_{j}} (t )\bigr\vert ^{n}}}= \frac{n!}{2^{{n}/{2}} ({n}/{2} )!} \bigl(\overline{\mathfrak {N}_{i}^{2}} (t ) \bigr)^{{n}/{2}}. $$

Again, since the neurons are all-to-all connected, the variance $\overline{\mathfrak{N}_{i}^{2}} (t )$ is the same for every neuron. Now, since

$$\frac{\overline{\mathfrak{N}_{i} (t )\mathfrak {N}_{j} (t )}}{\overline{\mathfrak{N}_{i}^{2}} (t )}=\operatorname{Corr}_{2} \bigl(V_{i} (t ),V_{j} (t ) \bigr) , $$

we obtain the following compact formula:

B.1 $$ \operatorname{Corr}_{n} \bigl(V_{i_{0}} (t ), \ldots ,V_{i_{n-1}} (t ) \bigr)= \textstyle\begin{cases} 0, & n\mbox{ odd}, \\ {} [\operatorname{Corr}_{2} (V_{i} (t ),V_{j} (t ) ) ]^{{n}/{2}}, & n\mbox{ even}, \end{cases} $$

where $\operatorname{Corr}_{2} (V_{i} (t ),V_{j} (t ) )$ is given by (4.10) and (6.15).

We stress the fact that having zero correlation for *n* odd is a consequence of the normal distribution of our three sources of randomness. For non-normal distributions this result is not true anymore. For *n* even, Eq. (B.1) proves that for the complete graph the higher-order correlations depend only on *n* and the pairwise correlation. This in general may not be true for other kinds of topologies. Moreover, since $\operatorname{Corr}_{2} (V_{i} (t ),V_{j} (t ) )\in [-1,1 ]$, then $\operatorname{Corr}_{n} (V_{i_{0}} (t ),\ldots ,V_{i_{n-1}} (t ) )\in [-1,1 ]$ as well, therefore the higher-order correlations are correctly normalized, as it must be.

## Footnotes

1 For completeness, it is worth clarifying that independence is a special case of a more general condition that mathematicians call *chaos*. Let $\nu_{t}$ be a probability measure on $\mathbb{R}^{d}$ at time *t*. We suppose this space is spanned by a vector variable **x**, which describes the state of a neuron. Then let $\mu_{t}^{ (N )}$ be a sequence of symmetric probability measures on $\mathbb {R}^{d\times N}$, where *N* is the number of neurons in the system. The sequence $\mu _{t}^{ (N )}$ is said to be $\nu_{t}$*-chaotic* if, for all integers $k\geq1$ and for all continuous and bounded test functions $\varphi_{1},\ldots ,\varphi_{k}$, we have

$$\begin{aligned} &\lim_{N\rightarrow\infty}\int_{\mathbb{R}^{d\times N}}\varphi_{1} (\mathbf {x}_{1} )\cdots\varphi _{k} ( \mathbf {x}_{k} )\,d\mu_{t}^{ (N )} ( \mathbf {x}_{1},\ldots, \mathbf {x}_{N} ) \\ &\quad= \biggl( \int_{\mathbb{R}^{d}}\varphi_{1} (\mathbf {x}_{1} )\,d\nu _{t} (\mathbf {x}_{1} ) \biggr)\cdots \biggl( \int_{\mathbb{R}^{d}}\varphi_{k} (\mathbf {x}_{k} )\,d\nu _{t} (\mathbf {x}_{k} ) \biggr) \end{aligned}$$

If the sequence $\mu_{t}^{ (N )}$ is $\nu_{t}$-chaotic, the neurons are said to be *chaotic* at time *t*. Intuitively, we can think of $\mu_{t}^{ (N )}$ as the joint probability of *N* (exchangeable) neurons. The exchangeability condition is equivalent to the above symmetry condition. We see that the case of independent and identically distributed neurons is a special one. Indeed, if $\mu _{t}^{ (N )}=\nu_{t}^{\otimes N}$ (where ⊗ denotes the tensor product of measures), then it is obvious that the above condition is satisfied ∀*N*. In turn, the tensor product of measures can be interpreted as the factorization of the joint probability that defines independence in probability theory. The initial conditions are said to be $\nu_{0}$-chaotic if the previous condition holds at time $t=0$. The propagation of chaos refers to the fact that if the initial conditions are $\nu_{0}$-chaotic, then, if the neurons are exchangeable, their joint law $\mu_{t}^{ (N )}$ is $\nu_{t}$-chaotic for some probability measure $\nu_{t}$ on $\mathbb{R}^{d}$ for all times $t\in [0,T ]$.

## References

[1] Womelsdorf T, Schoffelen J-M, Oostenveld R, Singer W, Desimone R, Engel AK, Fries P (2007). Modulation of neuronal interactions through neuronal synchronization. Science.

[2] Friston KJ (2011). Functional and effective connectivity: a review. Brain Connect.

[3] Sporns O, Chialvo D, Kaiser M, Hilgetag C (2004). Organization, development and function of complex brain networks. Trends Cogn Sci.

[4] Ponten SC, Daffertshofer A, Hillebrand A, Stam CJ (2010). The relationship between structural and functional connectivity: graph theoretical analysis of an EEG neural mass model. NeuroImage.

[5] Koch M (2002). An investigation of functional and anatomical connectivity using magnetic resonance imaging. NeuroImage.

[6] Eickhoff SB, Jbabdi S, Caspers S, Laird AR, Fox PT, Zilles K, Behrens TEJ (2010). Anatomical and functional connectivity of cytoarchitectonic areas within the human parietal operculum. J Neurosci.

[7] Cabral J, Hugues E, Kringelbach ML, Deco G (2012). Modeling the outcome of structural disconnection on resting-state functional connectivity. NeuroImage.

[8] Deco G, Ponce-Alvarez A, Mantini D, Romani GL, Hagmann P, Corbetta M (2013). Resting-state functional connectivity emerges from structurally and dynamically shaped slow linear fluctuations. J Neurosci.

[9] Hopfield JJ (1984). Neurons with graded response have collective computational properties like those of two-state neurons. Proc Natl Acad Sci USA.

[10] David O, Cosmelli D, Friston KJ (2004). Evaluation of different measures of functional connectivity using a neural mass model. NeuroImage.

[11] Sznitman A (1984). Nonlinear reflecting diffusion process, and the propagation of chaos and fluctuations associated. J Funct Anal.

[12] Sznitman A (1986). A propagation of chaos result for Burgers’ equation. Probab Theory Relat Fields.

[13] Sznitman A, Hennequin P-L (1991). Topics in propagation of chaos. Ecole d’eté de probabilités de saint-flour XIX – 1989.

[14] Tanaka H (1978). Probabilistic treatment of the Boltzmann equation of Maxwellian molecules. Probab Theory Relat Fields.

[15] Tanaka H (1981). Central limit theorem for a simple diffusion model of interacting particles. Hiroshima Math J.

[16] Tanaka H, Kallianpur G (1983). Some probabilistic problems in the spatially homogeneous Boltzmann equation. Theory and application of random fields.

[17] McKean H (1966). A class of Markov processes associated with nonlinear parabolic equations. Proc Natl Acad Sci USA.

[18] McKean H (1967). Propagation of chaos for a class of non-linear parabolic equations. Stochastic differential equations (Lecture series in differential equations, session 7, Catholic University, 1967).

[19] Samuelides M, Cessac B (2007). Random recurrent neural networks dynamics. Eur Phys J Spec Top.

[20] Touboul J, Hermann G, Faugeras O (2012). Noise-induced behaviors in neural mean field dynamics. SIAM J Appl Dyn Syst.

[21] Baladron J, Fasoli D, Faugeras O, Touboul J (2012). Mean-field description and propagation of chaos in networks of Hodgkin–Huxley and Fitzhugh–Nagumo neurons. J Math Neurosci.

[22] Touboul J (2014). The propagation of chaos in neural fields. Ann Appl Probab.

[23] Pernice V, Staude B, Cardanobile S, Rotter S (2011). How structure determines correlations in neuronal networks. PLoS Comput Biol.

[24] Pernice V, Staude B, Cardanobile S, Rotter S (2012). Recurrent interactions in spiking networks with arbitrary topology. Phys Rev E.

[25] Trousdale J, Hu Y, Shea-Brown E, Josić K (2012). Impact of network structure and cellular response on spike time correlations. PLoS Comput Biol.

[26] Ginzburg I, Sompolinsky H (1994). Theory of correlations in stochastic neural networks. Phys Rev E.

[27] Renart A, De La Rocha J, Bartho P, Hollender L, Parga N, Reyes A, Harris KD (2010). The asynchronous state in cortical circuits. Science.

[28] Bressloff PC (2010). Stochastic neural field theory and the system-size expansion. SIAM J Appl Math.

[29] Buice MA, Chow CC (2013). Dynamic finite size effects in spiking neural networks. PLoS Comput Biol.

[30] Faugeras O, MacLaurin J (2013). A large deviation principle for networks of rate neurons with correlated synaptic weights. BMC Neurosci.

[31] Faugeras O, Maclaurin J (2014). Asymptotic description of stochastic neural networks. I. Existence of a large deviation principle. C R Math.

[32] Faugeras O, Maclaurin J (2014). Asymptotic description of stochastic neural networks. II. Characterization of the limit law. C R Math.

[33] Ditlevsen S, Samson A, Bachar M, Batzel J, Ditlevsen S (2013). Introduction to stochastic models in biology. Stochastic biomathematical models.

[34] Bachar M, Batzel J, Ditlevsen S (2013). Stochastic biomathematical models: with applications to neuronal modeling.

[35] Magnus W (1954). On the exponential solution of differential equations for a linear operator. Commun Pure Appl Math.

[36] Isserlis L (1918). On a formula for the product-moment coefficient of any order of a normal frequency distribution in any number of variables. Biometrika.

[37] Chai B, Walther D, Beck D, Fei-Fei L, Bengio Y, Schuurmans D, Lafferty JD, Williams CKI, Culotta A (2009). Exploring functional connectivities of the human brain using multivariate information analysis. Advances in neural information processing systems 22.

[38] Thatcher RW, Krause PJ, Hrybyk M (1986). Cortico-cortical associations and EEG coherence: a two-compartmental model. Electroencephalogr Clin Neurophysiol.

[39] Honey CJ, Kötter R, Breakspear M, Sporns O (2007). Network structure of cerebral cortex shapes functional connectivity on multiple time scales. Proc Natl Acad Sci USA.

[40] Besserve M, Schölkopf B, Logothetis NK, Panzeri S (2010). Causal relationships between frequency bands of extracellular signals in visual cortex revealed by an information theoretic analysis. J Comput Neurosci.

[41] Sato JR, Junior EA, Takahashi DY, De Maria FM, Brammer MJ, Morettin PA (2006). A method to produce evolving functional connectivity maps during the course of an fMRI experiment using wavelet-based time-varying granger causality. NeuroImage.

[42] Bosman C, Schoffelen J-M, Brunet N, Oostenveld R, Bastos A, Womelsdorf T, Rubehn B, Stieglitz T, De Weerd P, Fries P (2012). Attentional stimulus selection through selective synchronization between monkey visual areas. Neuron.

[43] Barnett L, Seth AK (2014). The MVGC multivariate Granger causality toolbox: a new approach to Granger-causal inference. J Neurosci Methods.

[44] Tee GJ (2007). Eigenvectors of block circulant and alternating circulant matrices. NZ J Math.

[45] Boucsein C, Nawrot MP, Schnepel P, Aertsen A (2011). Beyond the cortical column: abundance and physiology of horizontal connections imply a strong role for inputs from the surround. Front Neurosci.

[46] Brouwer AE, Haemers WH (2011). Spectra of graphs.

[47] Munarini E, Perelli Cippo C, Scagliola A, Zagaglia Salvi N (2008). Double graphs. Discrete Math.

[48] Hansel D, Sompolinsky H, Koch C, Segev I (1998). Modeling feature selectivity in local cortical circuits.

[49] Genz A (1992). Numerical computation of multivariate normal probabilities. J Comput Graph Stat.

[50] Mazzoni A, Panzeri S, Logothetis NK, Brunel N (2008). Encoding of naturalistic stimuli by local field potential spectra in networks of excitatory and inhibitory neurons. PLoS Comput Biol.

[51] Shea-Brown E, Josić K, De La Rocha J, Doiron B (2008). Correlation and synchrony transfer in integrate-and-fire neurons: basic properties and consequences for coding. Phys Rev Lett.

[52] Quiroga RQ, Panzeri S (2009). Extracting information from neuronal populations: information theory and decoding approaches. Nat Rev Neurosci.

[53] Cavallari S, Panzeri S, Mazzoni A (2014). Comparison of the dynamics of neural interactions in integrate-and-fire networks with current-based and conductance-based synapses. Front Neural Circuits.

[54] Pillai SU, Suel T, Cha S (2005). The Perron–Frobenius theorem: some of its applications. IEEE Signal Process Mag.

[55] Renart A, Moreno-Bote R, Wang X-J, Parga N (2007). Mean-driven and fluctuation-driven persistent activity in recurrent networks. Neural Comput.

[56] Tetzlaff T, Helias M, Einevoll GT, Diesmann M (2012). Decorrelation of neural-network activity by inhibitory feedback. PLoS Comput Biol.

[57] De La Rocha J, Doiron B, Shea-Brown E, Josić K, Reyes A (2007). Correlation between neural spike trains increases with firing rate. Nature.

[58] Ecker AS, Berens P, Cotton RJ, Subramaniyan M, Denfield GH, Cadwell CR, Smirnakis SM, Bethge M, Tolias AS (2014). State dependence of noise correlations in macaque primary visual cortex. Neuron.

[59] Goris RL, Movshon JA, Simoncelli EP (2014). Partitioning neuronal variability. Nat Neurosci.

[60] Frank TD, Beek PJ (2001). Stationary solutions of linear stochastic delay differential equations: applications to biological systems. Phys Rev E.

[61] Yi S, Ulsoy AG (2006). Solution of a system of linear delay differential equations using the matrix Lambert function. Proceedings of the American control conference.

[62] Wormald NC, Lamb J, Preece D (1999). Models of random regular graphs. Surveys in combinatorics, 1999.

[63] Wilson HR, Cowan JD (1972). Excitatory and inhibitory interactions in localized populations of model neurons. Biophys J.

[64] FitzHugh R (1961). Impulses and physiological states in theoretical models of nerve membrane. Biophys J.

[65] Nagumo J, Arimoto S, Yoshizawa S (1962). An active pulse transmission line simulating nerve axon. Proc Inst Radio Eng.

[66] Hodgkin AL, Huxley AF (1952). A quantitative description of membrane current and its application to conduction and excitation in nerve. J Physiol.

[67] Lapicque L (1907). Recherches quantitatives sur l’excitation électrique des nerfs traitée comme une polarization. J Physiol Pathol Gén.

[68] Campbell SR, Wang DL (1996). Synchronization and desynchronization in a network of locally coupled Wilson–Cowan oscillators. IEEE Trans Neural Netw.

[69] Ledoux E, Brunel N (2011). Dynamics of networks of excitatory and inhibitory neurons in response to time-dependent inputs. Front Comput Neurosci.

[70] Macke JH, Opper M, Bethge M (2011). Common input explains higher-order correlations and entropy in a simple model of neural population activity. Phys Rev Lett.

[71] Montani F, Ince RAA, Senatore R, Arabzadeh E, Diamond ME, Panzeri S (2009). The impact of high-order interactions on the rate of synchronous discharge and information transmission in somatosensory cortex. Philos Trans R Soc A, Math Phys Eng Sci.

[72] Granot-Atedgi E, Tkac̆ik G, Segev R, Schneidman E (2013). Stimulus-dependent maximum entropy models of neural population codes. PLoS Comput Biol.

[73] Rowlinson P (1988). On angles and perturbations of graphs. Bull Lond Math Soc.

[74] Rowlinson P, Keedwell AD (1991). Graph perturbations. Surveys in combinatorics, 1999.

[75] Rowlinson P (1990). More on graph perturbations. Bull Lond Math Soc.

[76] Rowlinson P (1996). The characteristic polynomials of modified graphs. Discrete Appl Math.

[77] Cvetković DM, Rowlinson P, Simić S (1997). Eigenspaces of graphs.

[78] Van Den Heuvel MP, Sporns O (2011). Rich-club organization of the human connectome. J Neurosci.

[79] Mantegna RN (1999). Hierarchical structure in financial markets. Eur Phys J B.

[80] Sokal RR, Michener CD (1958). A statistical method for evaluating systematic relationships. Univ Kans Sci Bull.

[81] Minai AA, Williams RD (1993). Original contribution: on the derivatives of the sigmoid. Neural Netw.

[82] Carlitz L (1959). Eulerian numbers and polynomials. Math Mag.

[83] Miller SJ (2008). An identity for sums of polylogarithm functions. Integers.

[84] Deeba EY, Rodriguez DM (1991). Stirling’s series and Bernoulli numbers. Am Math Mon.

[85] Wood D. The computation of polylogarithms. Canterbury (UK): Computing Laboratory, University of Kent; 1992. Report No.: 15-92.

[86] Adegoke K, Layeni O (2010). The higher derivatives of the inverse tangent function and rapidly convergent BBP-type formulas for pi. Appl Math E-Notes.

